# The highly buffered Arabidopsis immune signaling network conceals the functions of its components

**DOI:** 10.1371/journal.pgen.1006639

**Published:** 2017-05-04

**Authors:** Rachel A. Hillmer, Kenichi Tsuda, Ghanasyam Rallapalli, Shuta Asai, William Truman, Matthew D. Papke, Hitoshi Sakakibara, Jonathan D. G. Jones, Chad L. Myers, Fumiaki Katagiri

**Affiliations:** 1 Department of Plant and Microbial Biology, Microbial and Plant Genomics Institute, University of Minnesota, Twin-Cities, Saint Paul, Minnesota, United States of America; 2 Department of Plant-Microbe Interactions, Max Planck Institute for Plant Breeding Research, Cologne, Germany; 3 The Sainsbury Laboratory, Norwich Research Park, Norwich, United Kingdom; 4 Department of Computer Science and Engineering, University of Minnesota, Twin-Cities, Minneapolis, Minnesota, United States of America; 5 RIKEN Center for Sustainable Resource Science, Yokohama, Japan; University of California Davis, UNITED STATES

## Abstract

Plant immunity protects plants from numerous potentially pathogenic microbes. The biological network that controls plant inducible immunity must function effectively even when network components are targeted and disabled by pathogen effectors. Network buffering could confer this resilience by allowing different parts of the network to compensate for loss of one another’s functions. Networks rich in buffering rely on interactions within the network, but these mechanisms are difficult to study by simple genetic means. Through a network reconstitution strategy, in which we disassemble and stepwise reassemble the plant immune network that mediates Pattern-Triggered-Immunity, we have resolved systems-level regulatory mechanisms underlying the Arabidopsis transcriptome response to the immune stimulant flagellin-22 (flg22). These mechanisms show widespread evidence of interactions among major sub-networks—we call these sectors—in the flg22-responsive transcriptome. Many of these interactions result in network buffering. Resolved regulatory mechanisms show unexpected patterns for how the jasmonate (JA), ethylene (ET), phytoalexin-deficient 4 (PAD4), and salicylate (SA) signaling sectors control the transcriptional response to flg22. We demonstrate that many of the regulatory mechanisms we resolved are not detectable by the traditional genetic approach of single-gene null-mutant analysis. Similar to potential pathogenic perturbations, null-mutant effects on immune signaling can be buffered by the network.

## Author summary

To protect themselves from pathogens, plants detect pathogen attack and send this information through a signaling network that activates various immune responses. Pathogens secrete effectors that disable components of the immune signaling network. Thus, the function of the plant immune signaling network must be well buffered from effector perturbations. Not much is known about how such network buffering is achieved. This is partly because the effects of mutations in single network components are also well buffered by the network. To overcome this shortcoming of single-gene mutant analysis, we employed a network reconstitution strategy, in which four major signaling sectors were first disabled and then restored one by one to determine the functions of the signaling sectors and their interactions. We collected and analyzed transcriptome and hormone profiles from all possible states of network reconstitution along time courses after immune stimulation with bacterial flagellin. We discovered that the network is not a collection of independent pathways; rather, interactions among the sectors dominate network regulation of the transcriptome response, which explains the extensive network buffering observed. Consequently, apparent network mechanisms inferred based on single-gene mutant analysis were often different from the underlying mechanisms.

## Introduction

A major tenet of systems biology is that complex biological systems are more than the sum of their parts [1]. This literally means that non-additive interactions among system components are important (hereafter we use the term interaction to specifically denote non-additive interaction). Because of these interactions, the function of a single gene in a complex signaling network may not be reliably inferred from null-mutant analysis of the gene alone; its function may be buffered by those of gene(s) in some other part of the network. To correctly assign functions to system components, the interactions among these components must be quantified.

Buffering has two different biological sources [2,3]: (I) closely related gene family members with redundant function may buffer each other, and (II) unrelated biological functions may buffer each other’s contribution to overall network function. Here we focus on the second case, which we call network buffering. Network buffering has been explored systematically in yeast, where the contributions of genetic interactions to yeast growth have been documented on a genome-wide scale [4].

Plants are faced with a barrage of pathogen assaults during their lifetimes. Plants resist pathogen attacks using both preformed barriers [5,6] and inducible immunity; the latter resembles innate immunity in animals [7]. In principle, microbial pathogens can evolve much faster than their hosts, and thus the hosts appear to face a serious adaptive disadvantage. In plants, which lack adaptive immunity, a highly buffered immune signaling network could confer resilience against perturbations to network components, as pathogen effectors are known to target and disable plant immune signaling proteins [8]. Buffering conceals the identity of network components: when a component in a buffered network is disabled, the network can compensate to some degree for this loss.

The resilience of the plant immune signaling network is qualitatively different from the robustness of switch-like networks, such as those that control development. These other networks ensure high-fidelity execution of an internal program despite internal and external noise. The plant immune signaling network, in contrast, must deploy the right response intensity depending on the reliability of attack information and attack severity—a fundamentally quantitative response, rather than a switch-like one, as unnecessary immune responses carry a fitness cost for the plant [9]. The plant immune signaling network needs to buffer not only random environmental noise, but more importantly, component loss due to targeting by pathogen effectors. By concealing the immune network’s mechanisms from pathogens, network buffering may confer resilience to the plant against pathogen adaptation.

Network buffering conceals a network’s underlying mechanisms not only from potential pathogens but also from our attempts to study the network. For example, null mutant analysis of individual genes in such a network may result in incorrect mechanistic interpretations [10]. How do we correctly assign component function in the presence of network buffering? One approach is to fully remove a given network function through deep network perturbation, and then stepwise reconstitute the network using combinatorial perturbations [11,12]. That is, break the network to the point where it loses resilience and investigate how network resilience emerges during reconstitution of the network. This is a reductionist approach applied to analysis of an emergent property of a network. To avoid a “combinatorial explosion”, the network must contain only a small number of components. For large complex networks, like the network that mediates plant inducible immunity, one strategy is to reduce the network size using a summarized scale.

A broad-spectrum form of inducible immunity, Pattern-Triggered-Immunity (PTI) can be stimulated by treating a plant with conserved microbe-associated molecular patterns (MAMPs), such as a portion of bacterial flagellin (flg22) [13]. The network that mediates flg22-PTI in Arabidopsis is almost entirely controlled by a network comprised of four sub-networks: the signaling mediated by the plant hormones salicylate (SA), jasmonate (JA) and ethylene (ET) and by the major immune regulator, phytoalexin-deficient 4 (PAD4) [11]. We call these sub-networks signaling sectors. Each of these sectors can be interrupted by disabling a single biosynthetic or signaling gene: *dde2-2* removes the JA sector [14], *ein2-1* the ET sector [15], *pad4-1* the PAD4 sector [16], and *sid2-2* the SA sector [17]. We previously removed the JA-ET-PAD4-SA network *in planta* via a quadruple mutant *dde2 ein2 pad4 sid2* and reconstituted the network stepwise via 4 triple mutants, 6 double mutants, 4 single-gene mutants, and the wild type [11,18].

Here we collected transcriptome and hormone profiles of these combinatorial mutants along a detailed time course and quantified how interactions among the four major sectors JA, ET, PAD4 and SA regulate the dynamic plant transcriptome in response to flg22 treatment. We show that sector interactions, rather than individual functions, dominated the regulation of the transcriptome response, and that buffering was extensive in the network. We further demonstrated several specific cases where the functional mechanisms inferred from shallow network perturbation did not reflect the regulation of the flg22-responsive and JA-ET-PAD4-SA network-dependent transcriptome. Our analysis spotlights a complex biological network that resists a classical genetics approach, a network where single-gene mutant analysis sometimes leads to incomplete or incorrect descriptions of component functions. We demonstrate the effectiveness of a systems biology approach, network reconstitution, to elucidate dynamical component functions of the plant immune signaling network.

## Results and discussion

### Overview of data collection and analysis

We profiled leaves of 31–32 days old plants of 17 Arabidopsis genotypes 0, 1, 2, 3, 5, 9, and 18 h after flg22 treatment. Sample infiltration was staggered (S2 Text), so that samples were collected as close to the same time of day as was experimentally feasible, because of known influences of the circadian cycle on plant immunity [19]. The genotypes used were our 16 combinatorial mutants for the 4 sectors, and a null mutant lacking FLS2, the flg22 receptor. In each mutant at each time point, we profiled both the plant transcriptome via stranded mRNA Tag-Seq libraries [20], and a suite of 44 plant hormones and related compounds via liquid chromatography-mass spectrometry (LC-MS) [21,22], including free SA and free JA. Transcript levels described in this paper are log_2_-transformed transcript level values (see Materials and methods and S1 Text for details).

**Fig 1:**
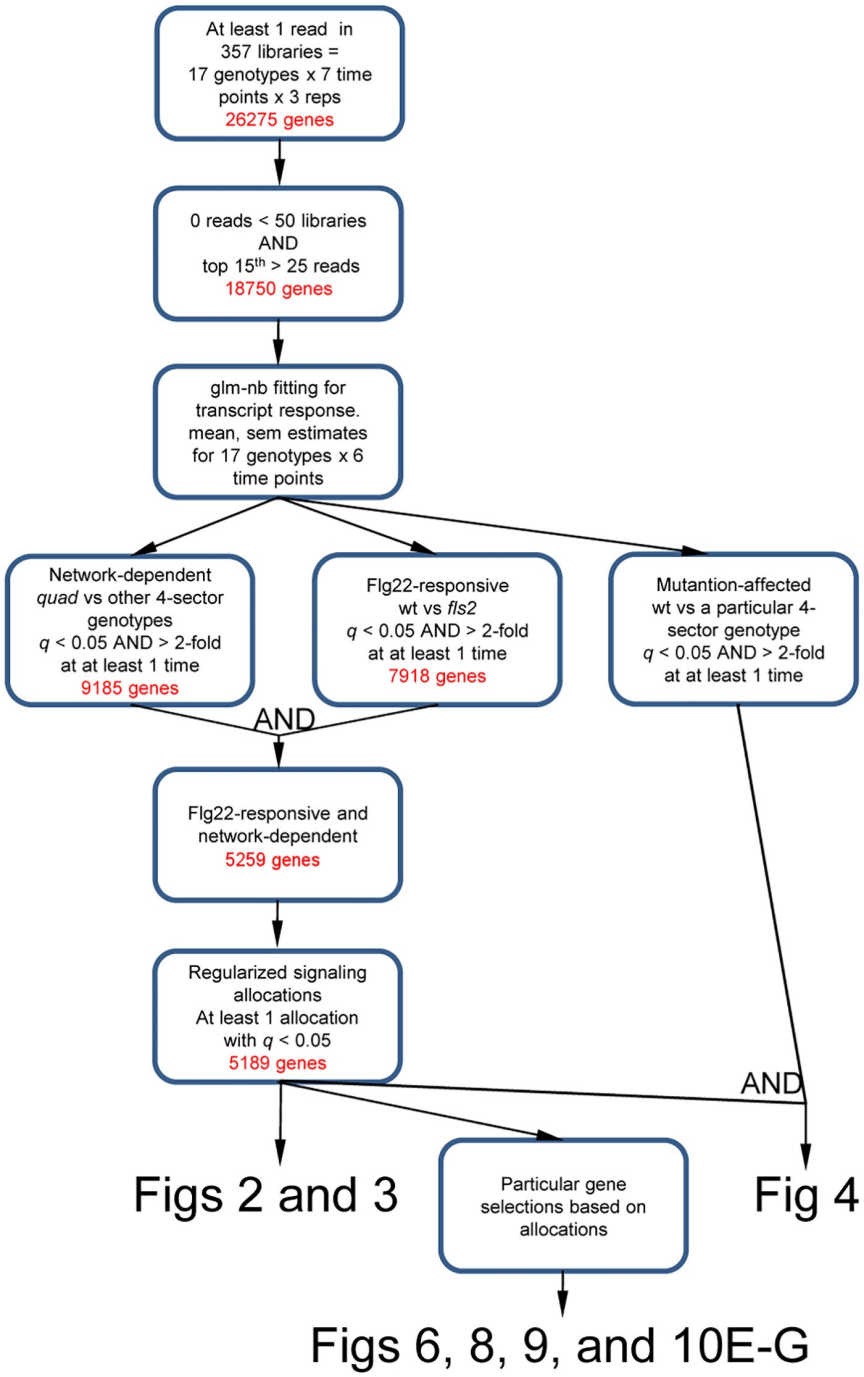
A summary of gene filtering procedures. Starting with 26275 genes that had at least 1 sequence read in at least one of the 357 3’-Tag-Seq libraries, this flowchart illustrates how genes used in network reconstitution analysis and Figs 2, 3, 4, 6, 8, 9 and 10E–10G were selected.

We selected genes and hormones for their dynamics and the sector effects on them. We focused on changes in transcript or hormone levels in response to flg22 treatment and did not consider network effects on basal transcript levels or basal hormone levels. To select transcripts and hormones to include in the analysis, we required that for at least one genotype and at least one time point, the transcript or hormone level response (relative to 0 hrs) was significantly different from the response in the quadruple mutant. This value is referred to hereafter as the transcript or hormone response change. We also required the transcript or hormone responses in wild type and *fls2* to be significantly different (flg22-specific response). The only hormone that passed these criteria was free SA. Free JA passed the hormone response change criteria but did not pass the flg22-specificity criterion as the free JA level also strongly responded in *fls2* (S1 Fig, panel B). Nonetheless, we included free JA in the subsequent analysis because many JA-responsive genes showed flg22-specific responses (see below) and because it is a well characterized immune hormone [23]. Among 5259 genes that passed these criteria, 5189 genes had significant signaling allocations (see below). We call these 5189 genes the flg22-responsive and network-dependent genes hereafter. There were approximately half as many flg22-responsive and network-independent genes in our dataset (2659 genes) as flg22-responsive and network-dependent genes (S2 Fig). In this report we focus on flg22-responsive and network-dependent genes since the JA-ET-PAD4-SA network is almost entirely responsible for flg22-triggered resistance [11]. The gene filtering procedures are summarized in Fig 1.

The transcript response change of each of the selected 5189 genes and 2 hormones was individually subjected to network reconstitution analysis to estimate both the sector effects and sector interactions that causally control the gene or hormone at each time point; these estimated effects and interactions are called signaling allocations [11,24]. The signaling allocation model for each gene or hormone for all time points was regularized by lasso [25,26] to remove insignificant terms. The stringency of the regularization was selected based on the corrected Akaike’s Information Criterion (AICc) [27,28]. The signaling allocation results with AICc-selected regularization stringency are used hereafter unless stated otherwise.

### The vast majority of the flg22-responsive and network-dependent genes showed complex network regulation

**Fig 2:**
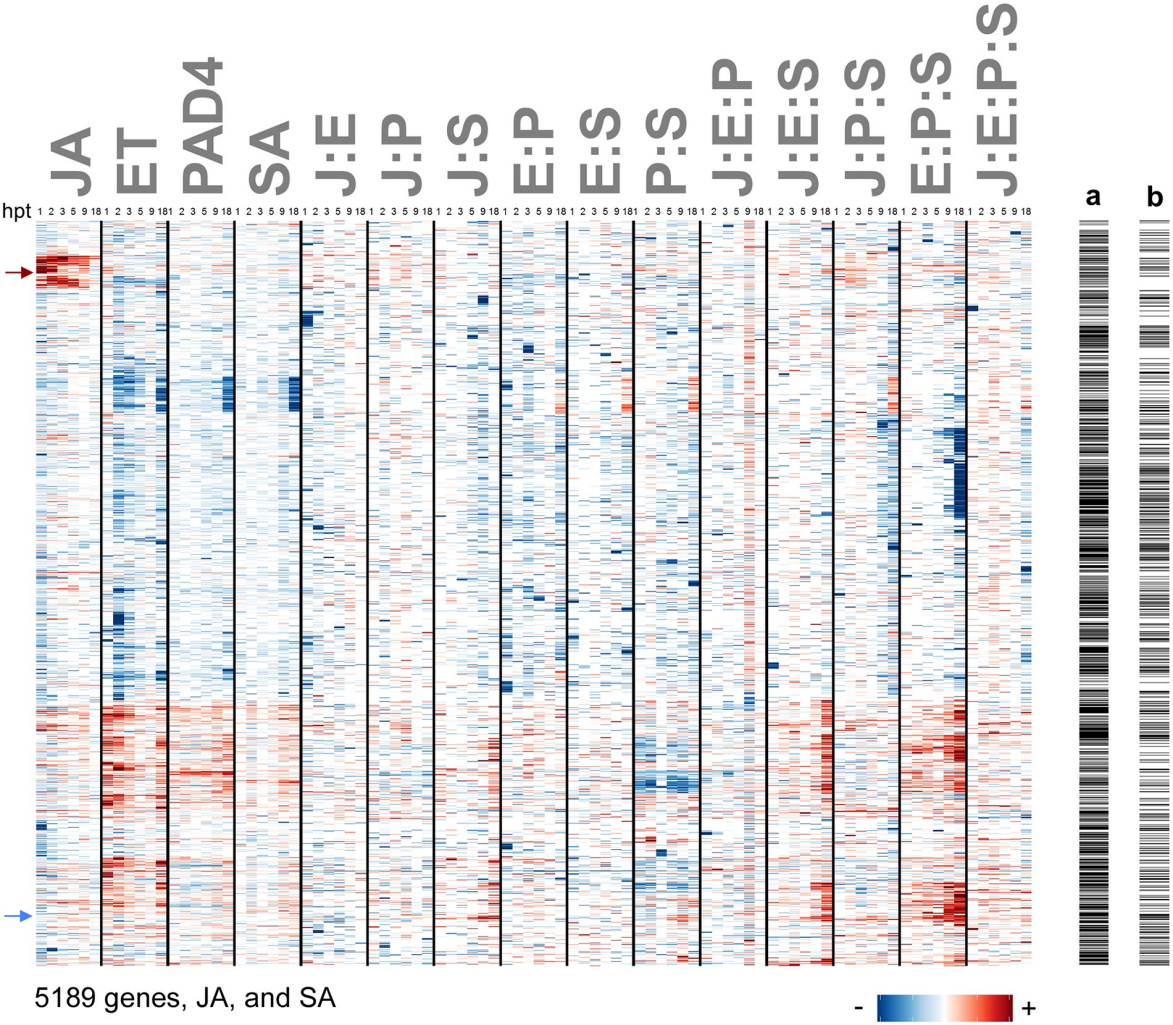
Network sector interactions are common in the regulation of the transcriptome response change to flg22 treatment. Note that even three- and four-sector interactions are common. A heatmap is shown for hierarchical clustering of 5189 flg22-responsive and network-dependent genes and the two hormones SA and JA (rows); clustering is based on their signaling allocations (columns). The signaling allocation model for each gene or hormone at all time points was regularized using lasso and the stringency of regularization was selected based on the AICc. The sign and magnitude of the signaling allocation contributions are indicated by color and intensity; red and blue for positive and negative, respectively; allocations removed by regularization are set to zero—visually they are white. Allocations were calculated for each gene or hormone from the transcript or hormone response changes through the time-series 1, 2, 3, 5, 9, and18 hours post treatment (hpt); 6 minor columns representing the 6 time points post treatment follow each other consecutively within each major column. Individual sector contributions are shown in the first four major columns and are indicated as JA, ET, PAD4, and SA. Sector interactions are shown in the rest of the major columns and indicated using ‘:’ with single letter notations of the sectors, for example the interaction between JA and ET is denoted ‘J:E’. Columns a and b on the right are indicator columns that mark, with black rows, (a) the 2979 genes whose transcript response relative to wild type is buffered in all single-sector mutants (*dde2*, *ein2*, *pad4*, and *sid2*), and (b) the 1564 genes whose transcript response relative to wild type is buffered in all single and double- sector mutants (*dde2*, *ein2*, *pad4*, *sid2*, *dde2 ein2*, *dde2 pad4*, *dde2 sid2*, *ein2 pad4*, *ein2 sid2*, and *pad4 sid2*). Red and blue arrows on the left of the heatmap indicate the rows corresponding to free JA and free SA, respectively. The 5189 flg22-responsive and network-dependent genes were selected according to the procedure described in the Materials and Methods. Signaling allocation rows were scaled for better visualization using the most extreme value of each row.

Of the 5189 genes and 2 hormones that showed a significant transcript (or hormone) response change, the signaling allocations of these response changes showed widespread evidence of complex network regulation (Fig 2). Signaling allocations are the linear decomposition of the transcript/hormone response relative to that of *quad* (transcript/hormone response change) into quantitative contributions of the individual sectors (JA, ET, PAD4, SA), and their interactions, which are indicated using single-letter abbreviations and ‘:’ (J:E, J:P, J:S, E:P, E:S, P:S, J:E:P, J:E:S, J:P:S, E:P:S, and J:E:P:S) (see S1 Text for mathematical details about fitting signaling allocation models to gene expression data). When two individual contributions have the same sign, and the interaction between them also has the same sign, the two sectors synergize to regulate the transcript response change. On the other hand, if two individual contributions have the same sign and the interaction between them has the opposite sign, the two sectors buffer each other (this is also referred to as compensation). There is no simple description of a particular interaction (such as synergy or buffering) when the individual contribution signs are different. Nevertheless, the signaling allocations provide a complete quantitative description for how the JA, ET, PAD4 and SA sectors regulate the response change of each transcript or hormone. As reported for pathogen growth data [11], the individual contributions of each sector to the transcript response changes in our dataset tended to have the same sign, that is, all four non-negative, or all four non-positive (Fig 2; all red or all blue, except for white, for the individual sector contributions). Among 5191 genes/hormones x 6 time points = 31146 gene:time combinations, 7533 gene:time combinations had more than one significant single sector contribution. Among the 7533 gene:time combinations, 22% of them were consistently non-negative, and 46% of them were consistently non-positive: in total 68% of the gene:time combinations had consistent signs across the single sector contributions. Given numerous literature cases that document antagonism between many of these sectors (JA and SA, JA and ET, SA and ET [29–33]), our analysis suggests that the source of these antagonisms is likely mediated by interactions for most of the flg22-responsive and network-dependent genes.

The robustness of the signaling allocation results was investigated by applying different stringencies of lasso regularization. S3 Fig panels A and B show the results with no regularization and with regularization whose stringency was selected based on Bayesian Information Criterion (BIC). The row orders of the genes and hormones in the heatmaps are the same in S3 Fig as in Fig 2. BIC-selected stringency is higher than the AICc-selected one, which was used for Fig 2, and use of BIC-selected stringency results in fewer non-zero terms in general. Major red and blue patterns in the heatmaps are well conserved at all three stringencies of regularization. This conservation is also clear when the non-zero signaling allocation values are compared among the unregularized, AICc-selected, and BIC-selected stringencies (S3 Fig panels C-E). This conservation of the patterns demonstrates the robustness of the signaling allocation results during regularization. It should be noted that regularization is not unbiased regarding the terms in the signaling allocation model. For example, the J:E:P:S interaction term is included only for the data from wild type, while the JA single sector contribution term is included for the data from eight genotypes that have the JA sector functional: i.e., the JA single sector contribution term tends to have a higher statistical power than the J:E:P:S interaction term. Therefore, lasso regularization tends to remove higher order interaction terms more than single sector contribution terms. Considering this bias in regularization, it is remarkable that major heatmap patterns conserved with the BIC-selected regularization stringency include several patterns in higher order interaction terms.

**Fig 3:**
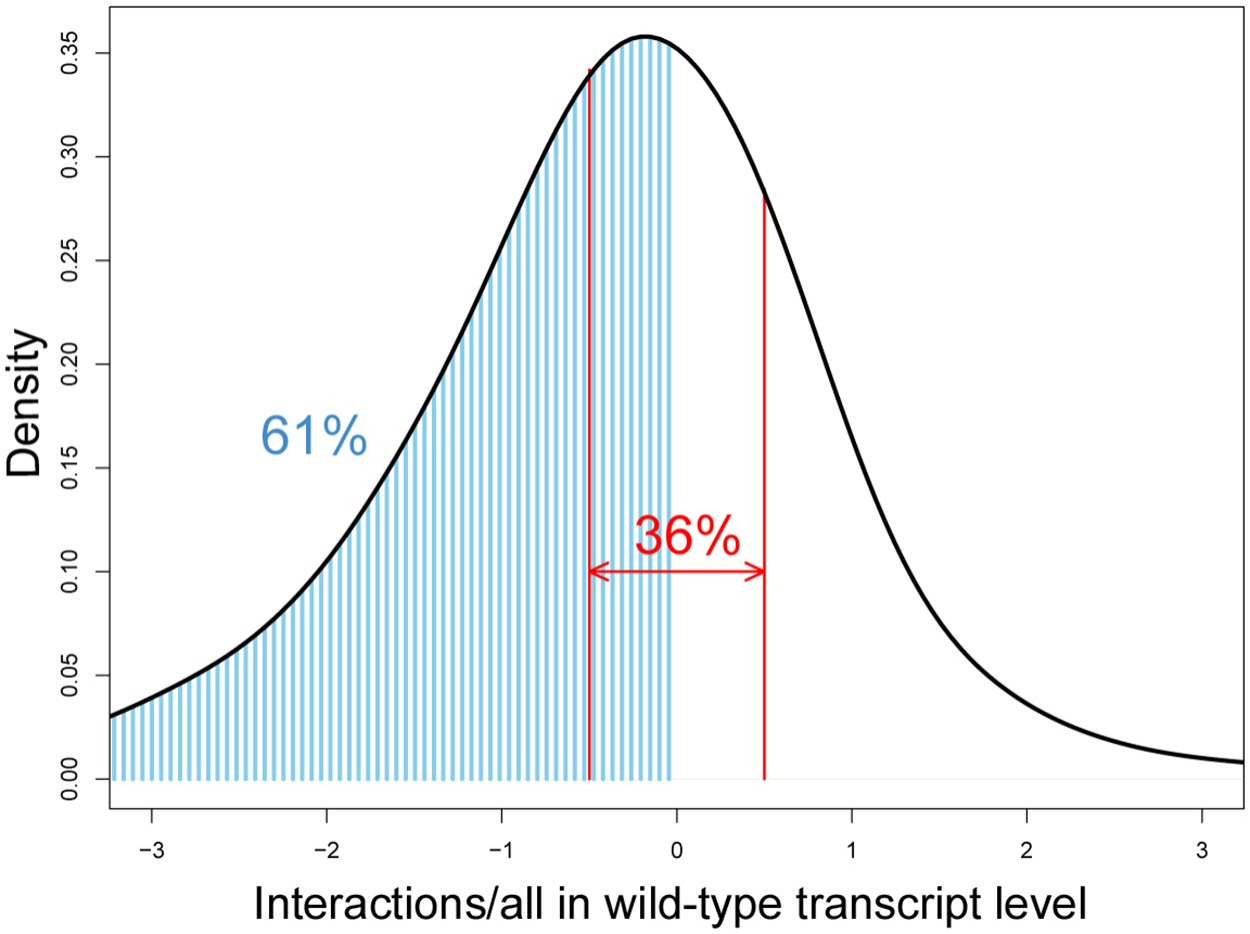
Network sector interactions control a major part of the wild-type transcript and hormone response changes. The relative net contribution of the sector interactions, which is the fraction of the summed sector interaction contributions in the wild-type transcript response change, was calculated for each gene/hormone at every time point. The cases in which the wild-type response change was less than 2-fold were omitted to avoid extreme errors in calculation of the fraction. The signaling allocation values from the unregularized model were used. The distribution of the calculated fractions is shown. When the sign of the interaction contribution is the same as or the opposite from that of the wild-type response change, the ratio has a positive or negative value, respectively. In 36% of the cases, the absolute value of the net interaction contribution is smaller than half of the wild-type response change (red arrow interval). In 61% of the cases, the net interaction contribution is negative (blue shaded area under the curve).

The bulk of the flg22-responsive and network-dependent transcriptome required substantial sector interactions to explain transcript behavior, including three- and four-sector interactions. Of the 5189 genes and two hormones, 99.5% had at least one interaction allocation after regularization. The relative net contribution of the sector interactions in the wild-type transcript response changes was evaluated. The relative net contribution of the interaction terms was calculated as the ratio of the value explained by all the sector interaction terms to the total transcript response change in wild type. Since there are cases, such as a large net positive contribution from the single sector terms and a large net negative contribution from the sector interaction terms for a positive transcript response change, the ratio value could take a value outside the range of [0,1], including a negative value. The value 0 indicates no net contribution from the sector interaction terms while the value 1 indicates no net contribution from the single sector terms. Fig 3 shows the distribution of the relative net contribution of the sector interactions across the genes/hormones at 6 time points. The results from the unregularized models (S3 Fig, panel A) were used in this analysis to avoid the bias against higher order interaction terms discussed above. Since the distribution is a compilation of the signaling allocations for all the genes and hormones at all time points, random errors in allocation results from the unregularized models should be averaged out in the distribution. Only in 36% of the cases was the absolute value of the net interaction contribution less than half of the wild-type expression response ([-0.5, 0.5], red arrow interval), indicating strong contributions of sector interactions in determining the wild-type response change. Sixty-one percent of the cases had negative net contributions of the interactions (i.e., an opposite sign from that of the wild-type transcript response change, blue shaded area under the curve), suggesting that network buffering is prevalent among the genes.

We have previously demonstrated that regulation of both a phenotypic output of this network (inhibition of pathogen growth), as well as core network activity (the regulation of four sector activity markers, one for each of the JA, ET, PAD4, and SA sectors), is complex [11,18]. Here we expand that observation, noting that transcript response changes of thousands of flg22-responsive and network-dependent genes were causally driven by interactions among the JA, ET, PAD4, and SA signaling sectors. Interactions among network components are an essential feature of any network that exhibits strong network buffering.

### More than half of the flg22-responsive and network-dependent genes are well buffered

**Fig 4:**
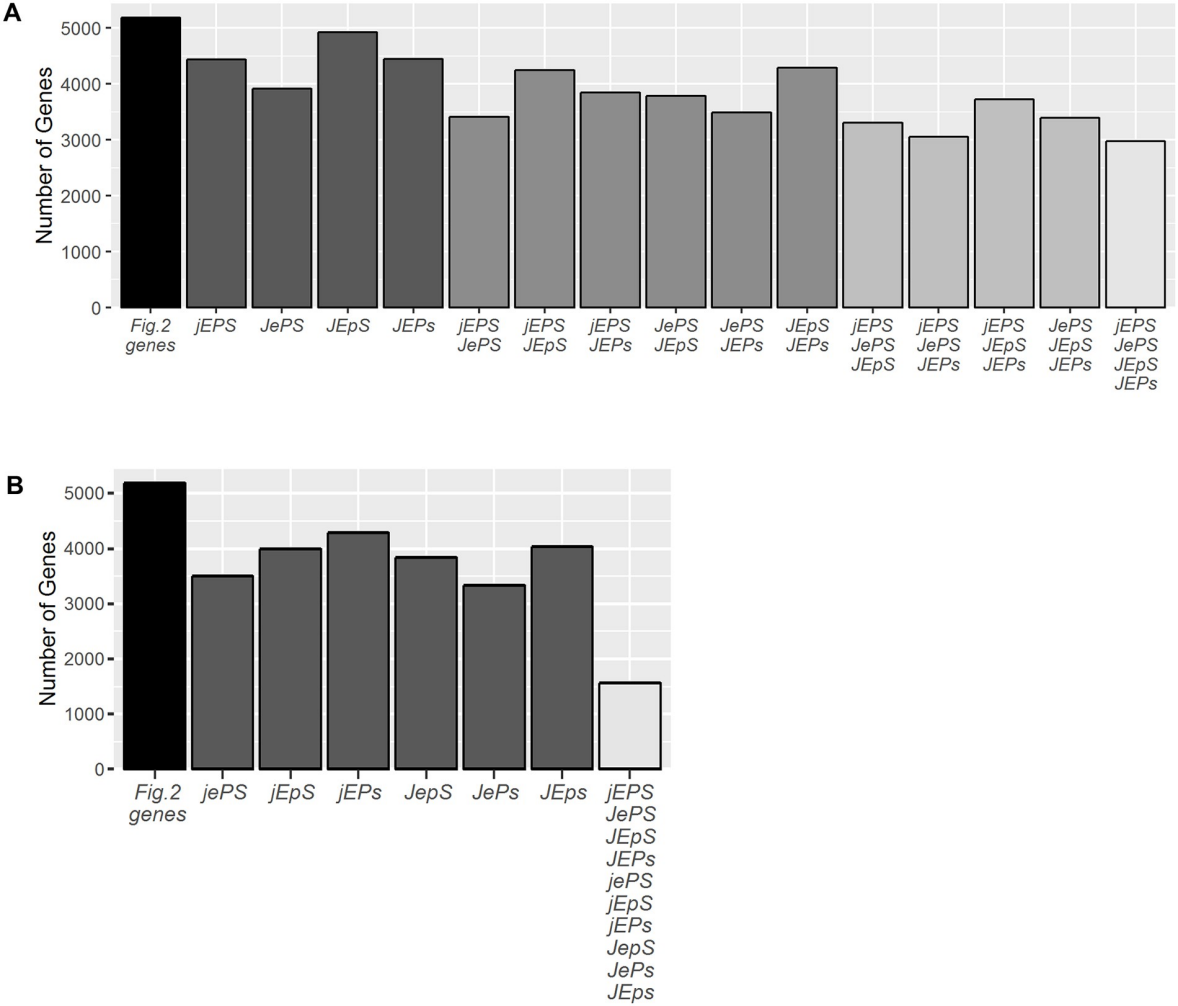
The responses of many genes to flg22 treatment are highly buffered by the network. (A) The number of the 5189 genes with buffered transcript response changes in each single-sector mutant, and in multiple single sector mutants. The first bar represents the genes in Fig 2; the last bar represents genes buffered in all single sector mutants (2977 genes). (B) The number of the 5189 genes with buffered transcript response changes in each double-sector mutant, and (last bar) the number of genes buffered in all single and double-sector mutants (1564 genes). Combinatorial sector genotypes are labeled using a concise genotype notation that indicates the presence (uppercase) or absence (lowercase) of the sectors JA, ET, PAD4, and SA. *dde2-2*, *ein2-1*, *pad4-1*, and *sid2-2* were used to remove the JA, ET, PAD4, and SA sectors, respectively. For example, the *dde2-2 pad4-1* double mutant is denoted as *jEpS*.

Among the 5189 flg22-responsive and network-dependent genes, 2977 genes were fully buffered—they showed no significant transcript response change relative to the wild type response in any single-sector mutant (black rows in Fig 2 column a indicate these 2977 genes; summary in Fig 4A); deeper network perturbations were required to show disturbances in network response. Furthermore, 1564 of these 2977 genes showed no significant transcript change in any double mutants either (black rows in Fig 2 column b indicate these 1564 genes; summary in Fig 4B). Yet these genes with clear network buffering had rich signaling allocation signatures (Fig 2). Thus, despite their lack of response changes in single- and double-sector mutant genotypes, these genes may be key players in plant immunity because they were clearly regulated by the network, each responded strongly to an immune stimulus (flg22), and these responses were highly buffered from potential pathogenic perturbations: they remained like those of wild type despite network perturbation. As the black rows in columns a and b in Fig 2 do not show obvious patterns, these genes strongly buffered are not associated with particular signaling allocation patterns. This indicates that the network includes multiple compensatory interactions that together buffer the transcriptome response as a whole.

### The four sectors show contrasting patterns of network dependence

**Fig 5:**
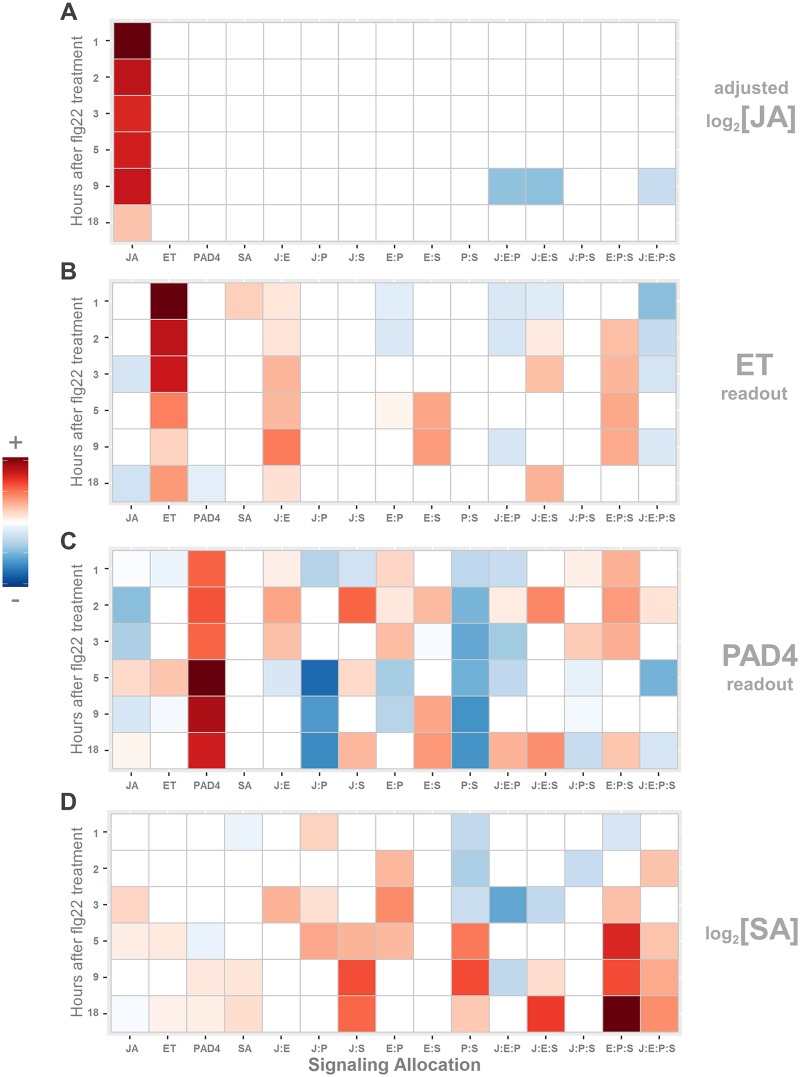
SA hormone induction completely depends on the other sectors. JA and ET hormone induction by flg22 is, on the other hand, mostly independent of the other sectors. Signaling allocations for responses of the following after flg22 treatment are shown: (A) adjusted log_2_ free JA hormone concentrations, (B) transcript levels of the ET sector marker gene *ARGOS*, (C) transcript levels of the PAD4 sector marker gene AT4G04500, (D) log_2_ free SA hormone concentrations. The notations for the signaling allocations are the same as those in Fig 2. The sign and magnitude of the signaling allocation contributions are indicated by color and intensity; red and blue for positive and negative, respectively; values for allocations removed by regularization are set to zero—visually they are white. Hormones and genes were individually scaled for better visualization using the most extreme value of each panel (sector). The time-course levels and responses of these sector activities in 17 genotypes are shown in S1 Fig.

**Fig 6:**
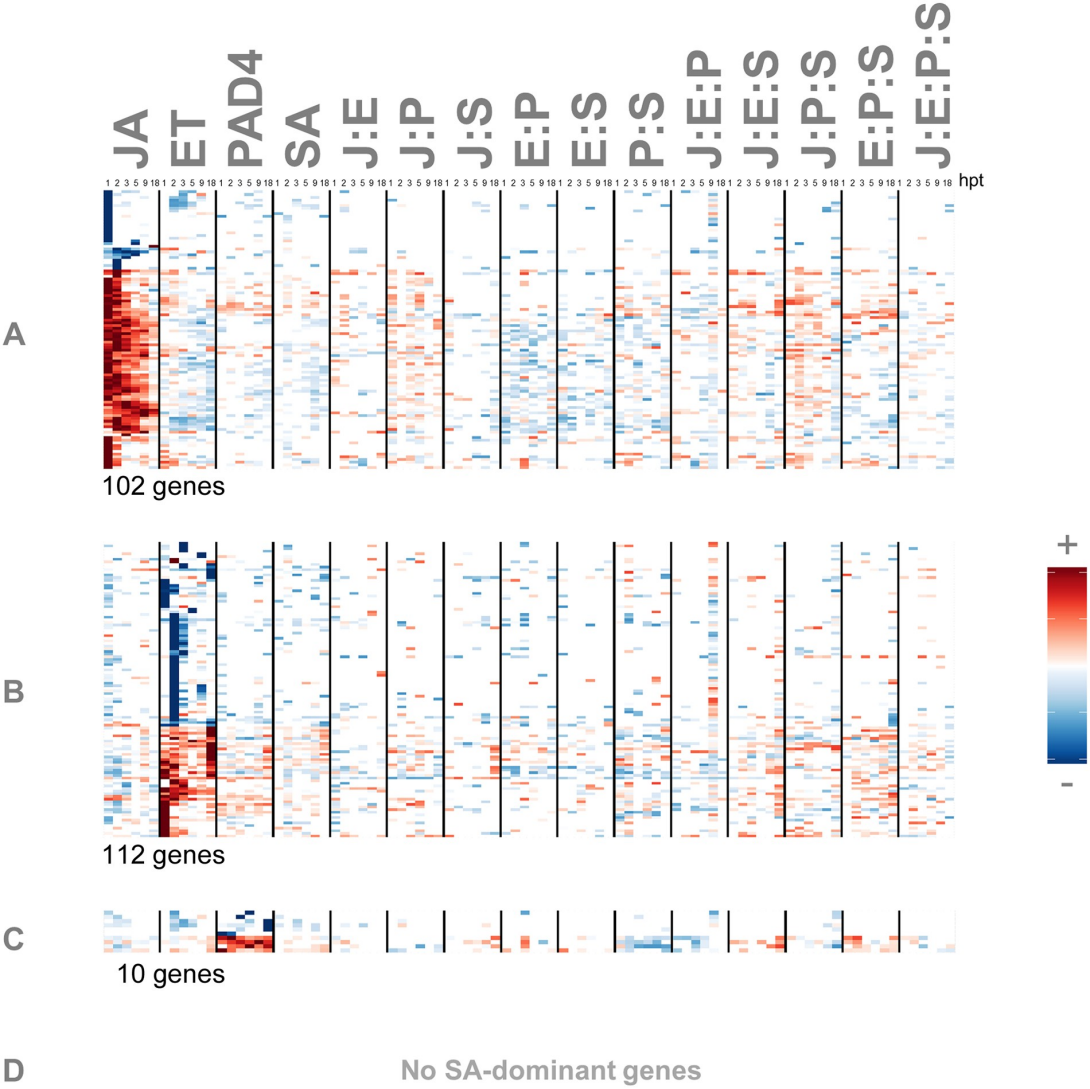
No genes are regulated by the SA sector alone. Genes whose transcript expression level responses are predominantly controlled by a single sector alone were selected for the (A) JA, (B) ET, (C) PAD4, and (D) SA sectors. For these genes, the maximum individual sector contribution is at least 1.6 times greater than all other contributions to each signaling allocation. The signs and magnitudes of the signaling allocation contributions are indicated by color and intensity; red and blue for positive and negative, respectively; insignificant values are set to zero—visually they are white. Allocations were calculated for each gene or hormone from the transcript or hormone response changes through the time series 1, 2, 3, 5, 9, and18 hours post treatment (hpt); 6 minor columns representing the 6 time points post treatment follow each other consecutively within each major column. The notations of the contribution labels are the same as those in Fig 2. Rows were individually scaled for better visualization using the most extreme value of each row.

While the major immune hormones, SA and JA, are both involved in the immune response to flg22 stimulus, the ways in which these hormones are activated and modulated by the rest of the JA-ET-PAD4-SA network are profoundly different. Induction of free JA hormone levels was nearly entirely dependent on only the JA individual sector contribution (Fig 5A). Dependence mainly on the JA sector is also seen as a pronounced signaling allocation pattern among the transcript responses: a set of 102 genes existed for which the main signaling allocation was JA alone (Fig 6A). In contrast, SA hormone levels could not be induced by flg22 independent of the other sectors; free SA was entirely dependent on the network for its induction (Fig 5D). Consistent with this, for no genes was the SA individual sector contribution the dominant signaling allocation (Fig 6D). Although there were seven genes that appeared to be in this category, these were likely all artifacts of too stringent regularization in the AICc-selected signaling allocation analysis since the unregularized results did not show any similar signaling allocation patterns (S4 Fig). There was no such obvious discrepancy in the allocation patterns between the AICc-selected and the unregularized for the genes selected for the JA, ET, or PAD4 sector alone (Fig 6A–6C).

Network regulations of the ET hormone response to flg22 resembled that of JA. Induction of ET hormone levels, estimated by the transcript level of the ET sector activity marker *ARGOS*, was nearly entirely dependent on the ET sector alone (Fig 5B). Similar results were seen with another ET sector activity marker (AT3G16530; S5 Fig, panel A). The transcript level of *ARGOS* is likely a reliable readout of the plant response to the ethylene hormone since (I) it showed no response in any *ein2*-containing genotypes (*ein2-1* abolishes ET response phenotypes [15]), (II) it is a direct target of the major transcriptional regulator of ethylene responses, *EIN3* [34], and (III) it responds strongly and quickly to exogenously applied ACC [35–37] (ACC is an ethylene biosynthetic precursor). As with JA, dependence mainly on the ET sector is a clear signaling allocation pattern among the transcript responses: 112 genes exist whose transcript response changes relative to the quadruple mutant response were almost entirely dependent on the ET individual contribution (Fig 6B).

Flg22 activation of the PAD4 sector showed a network dependence intermediate between those of SA and JA. PAD4 sector activation depended partly on the PAD4 individual sector contribution, but was also significantly affected by interactions among the sectors (Fig 5C). The PAD4 sector activity was monitored via the transcript level of a sector marker gene (AT4G04500) that passed strict criteria for abolished activity in *pad4*-containing genotypes. One striking feature of the PAD4 signaling allocation is strong negative P:S interactions (Fig 5C). This is because the main difference in the marker transcript levels between *jePs* and *jePS* is that *jePS* has a much higher basal level (0 hpt) while the induced levels are not that different (S1 Fig, panel E). Consequently, *jePS* has a much lower response than *jePs*. (S1 Fig, panel F), which is the reason for the strong negative P:S interactions. Another candidate PAD4 sector activity marker gene shows similar network dependence (S5 Fig, panel B). We used these sector activity gene candidates to monitor the PAD4 sector since no method is known to directly monitor the activity of the PAD4 protein.

**Fig 7:**
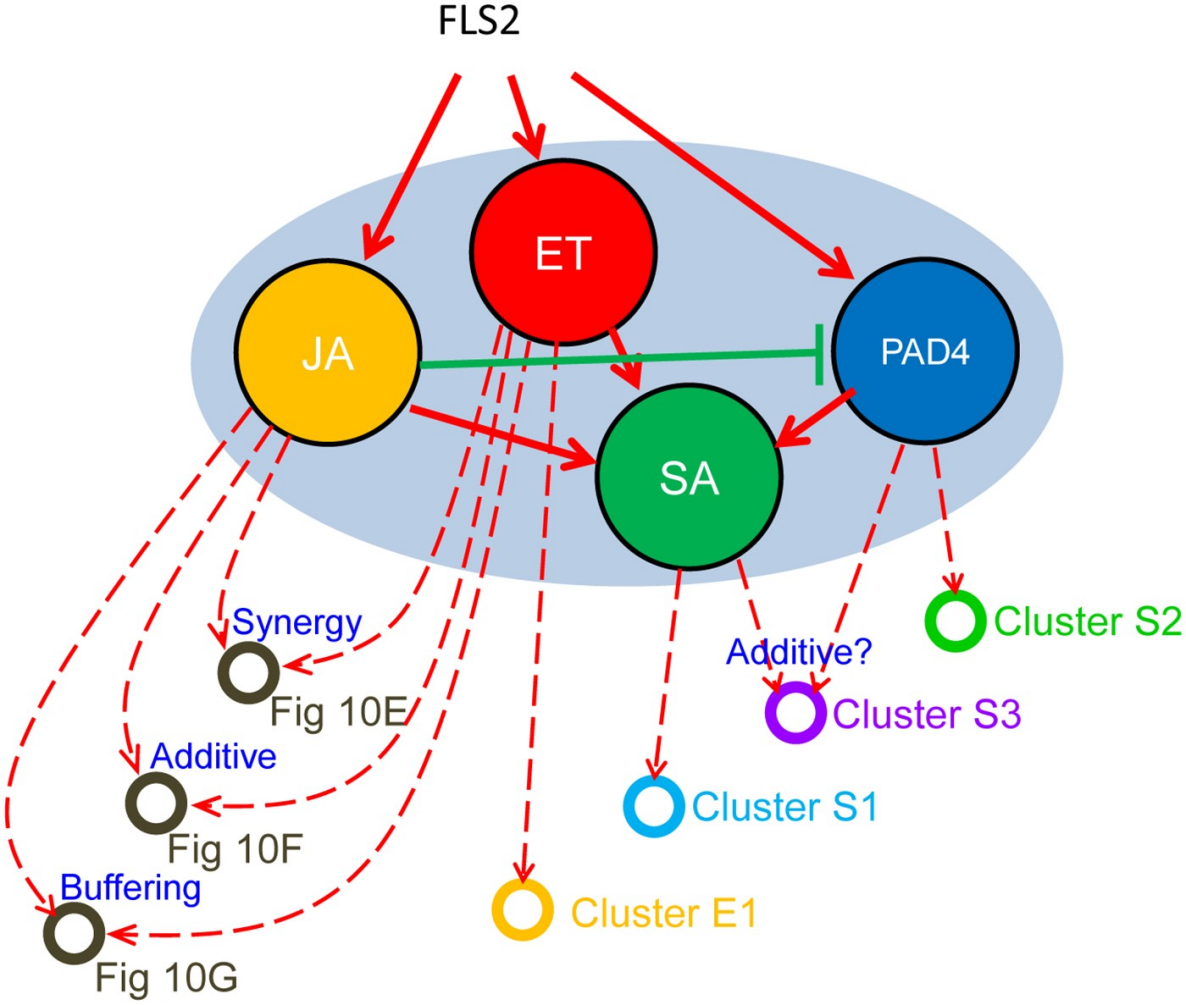
Signal interactions occur within the 4-sector network and downstream to the network. Some of the signal flows between the signaling sectors and signal interaction among the sector outputs are schematically depicted. The signal flows into the network and among the 4-sectors are shown by solid arrows in red (activation) and green (inhibition). Some output signal flows from the 4 sectors that regulate the transcript response are shown by dashed arrows in red (activation). The types of interactions are indicated at the arrow-converging nodes (synergy, additive, and buffering). The signaling allocations of the genes representing the nodes are shown in the indicated Figs or the clusters in Figs 8 and 9.

Activation of the four different summarized network sectors, JA, ET, PAD4, and SA, by flg22 treatment was largely consistent with our previous sector activity modeling results for MAMP-triggered PTI [18]. Those results are: the JA, ET, and PAD4 sectors are activated by flg22-responsive signaling from outside the network; flg22 activation of SA is entirely indirect—activation occurs through the other sectors of the network. These relationships are included in Fig 7. However, the diagram in Fig 7 does not contain information about interactions among multiple inputs to a signaling sector (i.e. information from higher-order interactions): e.g., how the inputs from the JA, ET, and PAD4 sectors interact in controlling the SA sector response. It should be noted that while this type of diagram explains signal flows between sectors in isolation from the other sectors, it has limited use in predicting behaviors of the intact network.

**Fig 8:**
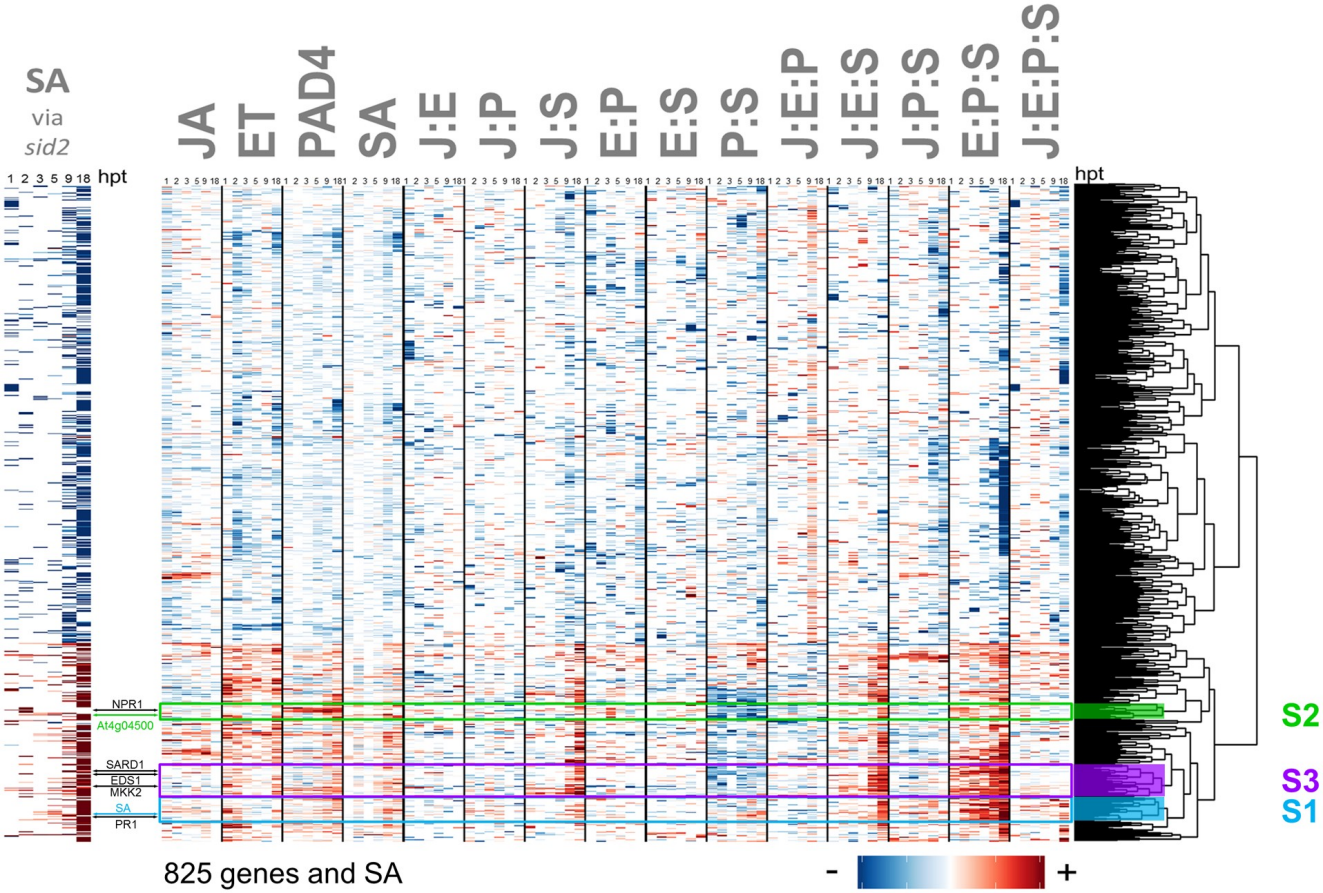
Network reconstitution reveals multiple regulatory mechanisms controlling expression of the traditionally-defined SA-dependent genes. Signaling allocations are shown for the 825 genes that had a significant transcript level change in *sid2* relative to the transcript level in wild type among the 5191 flg22-responsive, network-dependent genes/hormones. The left panel shows the strength of a traditionally-defined SA response: those with decreased and increased transcript levels in the single sector mutant compared to wild type are shown by red and blue bars, respectively. For genes that are positively regulated by SA according to this traditional definition (red rows in the left panel), the network reconstitution revealed multiple regulatory patterns, which include genes whose regulation closely followed that of SA (sky blue arrow), and thus were regulated almost entirely by network interactions (cluster S1, sky blue) and those that share a regulatory pattern with the PAD4 sector activity marker AT4G04500 (green arrow; cluster S2, green). Some of the genes whose expression is well known to be affected by SA, *NPR1*, *SARD1*, *EDS1*, *MKK2*, and *PR1*, are also indicated by black arrows. The cluster including *SARD1*, *EDS1*, and *MKK2* (cluster S3, purple) appears to be a mixture of characteristics of clusters S1 and S2. Cluster S3 genes may be mainly regulated by additive effects of the SA and PAD4 sectors. Although the *SID2* gene itself is very close to the bottom of the heatmap, below cluster S1, and the *PAD4* gene is slightly above cluster S2 in the heatmap, the data for the transcript levels of these genes cannot be properly interpreted in this dataset since each of these genes is mutated by design in half of the combinatorial genotypes.

Importantly, sector-interaction contributions to signaling allocations have two different sources: interactions within the 4-sector network, and those that occur downstream of the network (e.g., when signals from multiple sectors are integrated for regulation of some transcript responses, but this integrated signal is not fed back to influence the activity states of the sectors). For example, since free JA and the ET sector marker genes have simple signaling allocation signatures, a J:E interaction must involve signal integration downstream of the JA-ET-PAD4-SA network. For SA and PAD4, however, the sector responses are themselves regulated in a complex manner; interaction terms that include ‘S’ or ‘P’ could depend on interactions within the network, downstream of the network, or both. Note that genes that follow SA regulation (Fig 8 cluster S1) are driven by complex network interactions, and yet the mechanistic interpretation is simple: these genes largely respond to free SA (Fig 7).

### Network reconstitution revealed unexpected patterns of transcriptional and hormone regulation

Antagonism between SA and JA is widely reported [29–31]. In contrast to these observations, we previously reported that induction of SA absolutely requires the JA sector after flg22 treatment when the PAD4 sector is absent [18]. Here we report that, consistent with our previous discovery, both the SA hormone and the entire suite of genes whose regulation closely followed that of free SA (Fig 8, cluster S1) have strong positive J:S and J:E:S allocations at a later time point (18 hpt). We note that the positive effect of JA on SA was entirely buffered by the network: SA induction was slightly higher in the single-sector mutant lacking the JA sector (*dde2-2*) than in the wild type (S1 Fig, panel H: note that *jEPS* (yellow-green-blue line) is slightly higher than *JEPS* (black line; wild type)). By traditional genetic analysis, this would have led us to infer that JA inhibits SA induction, in agreement with the literature. However, network reconstitution revealed the mechanisms underlying SA activation. The PAD4 effect on SA inducibility is very similar to the JA effect on SA inducibility (S1 Fig panel H: the lines for *jePS* (green-blue) and *JepS* (red-blue) are very close), which is also seen as strongly positive J:S and P:S interactions in regulation of the SA sector response (Fig 5D). However, the JA sector strongly inhibits the PAD4 sector response, which is seen as a strongly negative J:P interaction in regulation of the PAD4 sector response (Fig 5C). As a consequence of this inhibition, when both the JA and PAD4 sectors are active in the network, the SA sector response is lower than when either the JA or PAD4 sector alone regulates the SA sector response (S1 Fig, panel H; the line for *JePS* (red-blue-green) is lower than those for *jePS* (green-blue) and *JepS* (red-blue)). Thus, this negative regulation of the PAD4 sector response by the JA sector explains why removal of the JA sector from the wild-type network resulted in a higher SA inducibility. An early study in *Nicotiana benthamiana* [38] reported that plants treated exogenously with SA and low concentrations of JA showed strong positive synergy between SA and JA in the accumulation of *PR1a* transcript and protein (*PR1a* is the tobacco homolog of the Arabidopsis *PR1* gene, whose transcript expression levels are widely used as a proxy for SA hormone concentrations; Fig 8, cluster S1). This early observation can be explained by assuming that in *N*. *benthamiana* at the concentration of JA used in the study, the inhibition of the PAD4 sector response by the JA sector activity is much weaker than in our conditions in Arabidopsis. There is a noteworthy difference between the effects of JA and PAD4 sectors on regulation of the SA sector activity: while the JA and PAD4 sectors have similar effects on the SA inducibility, only the PAD4 sector has a positive effect on the SA basal level (S1 Fig, panel G; *jePS* (green-blue line) is clearly higher than *jepS* (blue line) at 0 hpt whereas *JepS* (red-blue line) is not much higher than *jepS* (blue line) at 0 hpt). This exemplifies that the effects of the sectors could be different on the response and on the basal activity of a given sector or a gene. Further, it supports the notion that regulation of the response and the basal activity need to be separately described, as their mechanistic interpretations may be different.

*PAD4* is required for SA hormone accumulation after pathogen challenge [39,40]. Yet the interaction between PAD4 and SA sectors (P:S), was only a moderately positive contribution to the signaling allocation for the SA hormone levels (Fig 5D) and to the transcriptome cluster that closely followed SA (Fig 8, cluster S1). The visually dominant player was the high-order interaction E:P:S, meaning that the strong interaction between the PAD4 and SA sectors required ET sector function as well. Basal *FLS2* transcript levels (time, 0 h) were low in all *ein2*-containing mutants (S6 Fig), which is consistent with previous reports [41] and may explain this ET sector dependence in the E:P:S interaction. Nevertheless, there is a line of evidence that supports the notion that the FLS2 protein level that is potentially reduced by the ET sector removal does not have a strong influence on our observations. First, if the *ein2* mutation had a strong reduction in the signal transduced from FLS2, strong and general reductions in the flg22-specific transcriptome response would be expected in the *ein2*-containing genotypes. However, the impact of the *ein2* mutation on the flg22-specific transcriptome response is limited to a relatively small number of genes: 1270 *ein2*-affected genes among 5189 flg22-responsive, network-dependent genes (Fig 4A) plus 2659 flg22-responsive, network-independent genes (S2 Fig), which shows that the *ein2*-affected genes represent only 16% of all flg22-responsive genes. Among the 32 cluster S1 genes, 11 genes overlapped with the 1270 *ein2*-affected genes, indicating no enrichment of the *ein2*-affected genes in cluster S1 (*p* = 0.21, compared within the 5189 flg22-responsive, network-dependent genes). Second, the *FLS2* transcript levels fully recovered in all *ein2*-containing mutants by 1hr after flg22 treatment, our earliest post-treatment time point (S6 Fig). This observation suggests that the transcript levels of the genes that are affected by the *ein2* mutation through the FLS2 protein level would be more strongly affected at earlier time points. When the transcript responses of 100 randomly selected *ein2*-affected genes in *JEPS* (wild type) and *JePS* (*ein2* mutant) were plotted, no such expected time-dependent patterns were evident (S7 Fig). Third, activation of key kinases that mediate flg22 responses, MAP kinases *MPK3* and *6*, still occurs in the quadruple mutant at a level comparable to the wild-type 10 m after flg22 treatment [11]. We conclude that potentially reduced FLS2 protein levels in *ein2*-containing backgrounds did not have strong impacts under our experimental conditions, probably due to treatment with a high concentration of flg22 (1 μM). Thus, the observed strong E:P:S interaction likely represents a genuine property of the network that mediates signaling that ensues after FLS2 activation.

### Network reconstitution revealed complex regulatory mechanisms that were concealed from null-mutant analysis of the individual genes in the 4-sector network

Induction of SA in response to pathogens is absent in the *sid2-2* mutant [17] (*JEPs* (red-yellow-green line) in S1 Fig, panel H). As such, the *sid2-2* mutant, which we used to remove the SA sector, has been used in pathogen-responsive transcriptome profiling to define SA-dependent genes [42]. We found that the network regulation of SA-dependent genes that respond to flg22 stimulus could be classified into multiple clusters (Fig 8). The clusters include clusters S1 and S2: cluster S1 contains SA, and cluster S2 contains the PAD4 sector activity marker gene, AT4G04500. Cluster S1 is characterized by strongly positive E:P:S interactions, weak P:S interactions, and very weak single sector contributions while cluster S2 is characterized by weakly positive E:P:S interactions, strongly negative P:S interactions, and strongly positive PAD4 contributions. A canonical SA marker gene, *PR1*, is in cluster S1 while another SA-responsive gene, *NPR1*, is in cluster S2. These multiple clusters illustrate that the SA-dependent genes that are defined in a conventional manner are comprised of gene groups that are regulated differently. In fact, some of the well-known SA markers, such as *MKK2*, *EDS1*, and *SARD1*, which are characterized by very strongly positive J:S, J:E:S, and E:P:S interactions, are outside of clusters S1 and S2 (Fig 6, cluster S3). Network reconstitution, based on deep and combinatorial network perturbations, can not only detect regulatory differences among these genes but also assign the sources of differences to particular signaling sector contributions and their interactions.

**Fig 9:**
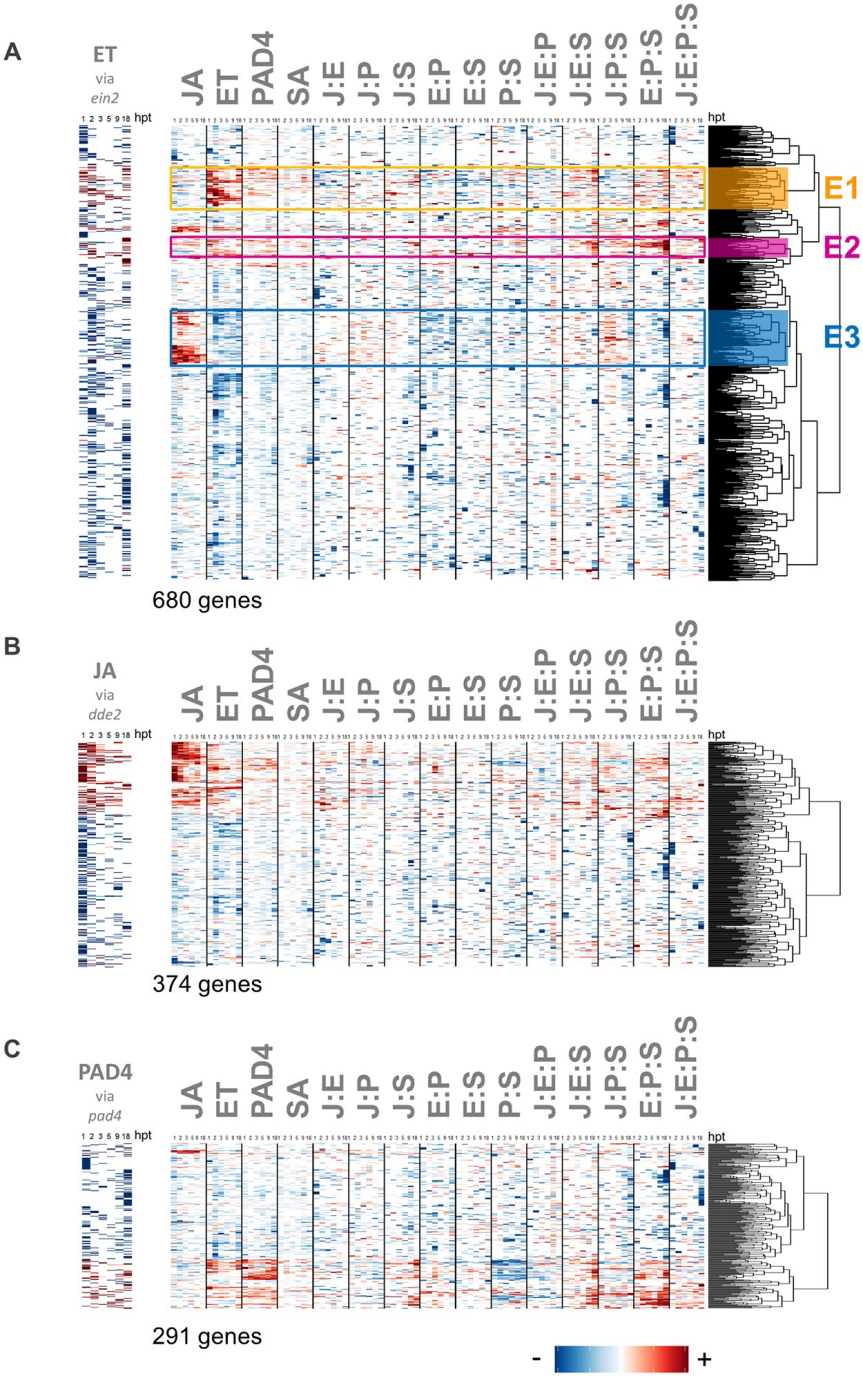
Regulation of genes strongly impacted by the ET, JA, and PAD4 single sector perturbations. Network reconstitution uncovers the regulation of genes whose flg22 transcript response changes are strongly impacted by the ET (A), JA (B), and PAD4 (C) single sector perturbations *dde2*, *ein2*, and *pad4*, respectively. Signaling allocations are shown for the 680 genes (A), 374 genes (B), and 291 genes (C) that had a significant transcript level response in *ein2* (A), *dde2* (B), and *pad4* (C) relative to the transcript response in wild type. The left panels show the strength of a traditionally-defined sector response: those with decreased and increased transcript levels in the single sector mutant compared to wild type are shown by red and blue bars, respectively. Three clusters (E1, E2 and E3) are indicated by orange, purple, and blue colors, respectively. Signaling allocation rows were scaled for better visualization using the most extreme value of each row.

Genes with significant transcript response changes in the ET single-sector mutant (*ein2-1*) also showed distinct signaling allocation patterns. Apparent regulation is based on the transcript response change in *ein2* compared to wild type (Fig 9A, the heatmap on the left). Many of the genes apparently regulated by ET were indeed regulated mainly by the ET sector alone (Fig 9A cluster E1). For a subset of genes apparently regulated positively by ET, however, the regulation relied on a suite of interaction-based signaling allocation terms, particularly the E:P:S interaction term (Fig 9A cluster E2). Although this pattern is reminiscent of the set of interactions involved in SA regulation, only one of the 21 genes (AT3G07340) overlapped with the 825 apparently SA-responsive genes in Fig 8. Among genes with apparently negative regulation by ET (Fig 9A, blue in the heatmap on the left), most were mainly negatively regulated by the ET sector, at earlier time points (Fig 9A), while for a distinct subset, the regulatory pattern was a strongly positive regulation by the JA sector, and only moderately negative regulation by the ET sector (Fig 9A cluster E3).

The majority of the genes apparently regulated positively by the JA sector (red rows in the left panel of Fig 9B) were indeed mainly regulated by the JA sector alone (Fig 9B, right panel). On the contrary, most of the genes apparently regulated negatively by the JA sector (blue rows in the left panel of Fig 9B) did not show obvious contributions from the JA sector (Fig 9B, right panel), indicating that the regulation of these genes is mainly dependent on sector interactions.

291 genes and SA showed significant transcript/hormone response changes in the *pad4-1* single-sector mutant relative to the wild type transcript response (Fig 9C). The observed signaling allocation patterns are similar to those selected with the *sid2* single mutant (Fig 8) although the number of selected genes for *pad4* is less than a half of that for *sid2*. In fact, among the 291 genes selected for *pad4*, 170 genes are also included in those for *sid2*: the overlap was highly significant (*p* < 2.2 x 10^-16^, Fisher’s exact test). The observation of a high level of overlap between the two gene sets confirms the well-coordinated regulatory relationships between the PAD4 and SA signaling sectors [43]. At the same time the observation that the gene set selected by *pad4* is smaller than that selected by *sid2* suggests that the PAD4 sector function is better buffered than the SA sector function.

### Concurrent activation of both the JA and ET sectors is not sufficient for induction of the genes in the ERF-branch of JA signaling

One well-studied example of immune hormone cross-talk in Arabidopsis is a branch of JA signaling that depends on positive synergy between JA and ET. This ERF-branch requires both JA and ET for full activity [44], and is regulated by ethylene response factor (ERF) transcription factors (TFs). ERF-branch activity is commonly monitored by the marker genes *PDF1*.*2a*, *PR3*, and *PR4* [45–49]. The ERF TFs are highly functionally redundant, making identification of ERF-branch genes by loss-of-function approaches difficult, but an overexpression line of the ERF TF gene *ORA59* was used to identify ERF-branch genes that are regulated by ERF TFs. By selecting within these genes for those that respond synergistically to exogenous JA and ethephon (an ethylene-releasing agent) [46], a set of putative ERF-branch genes was assembled.

**Fig 10:**
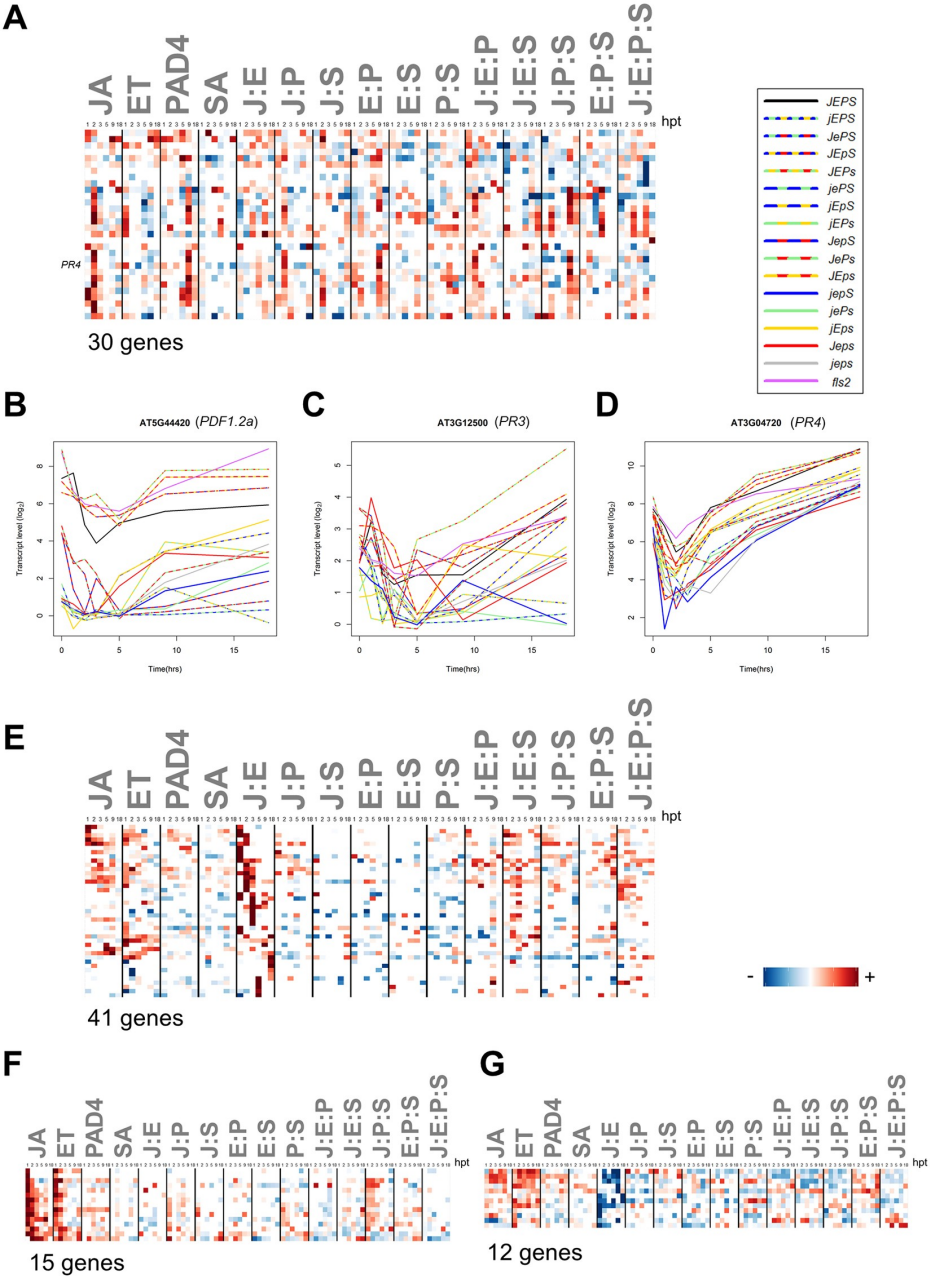
Regulation of the ERF-branch genes is not dominated by the J:E interaction. (A) Thirty putative ERF-branch genes, including *PR4*, do not share dominating signaling allocation patterns. Signaling allocation rows were scaled for better visualization using the extreme values of each row. (B-D) The time-course transcript levels of canonical ERF-branch marker genes, *PDF1*.*2a* (B), *PR3* (C), and *PR4* (D) in the 17 genotypes. Colors indicate genotype by presence of a sector: red (JA), yellow (ET), green (PAD4), blue (SA). Other genotypes: black (wild type), gray (quadruple mutant), purple (*fls2*). The entire color code is shown at the right of panel (A). (E-G) There are genes with strongly positive J:E interactions (E; 41 genes), with positive JA and ET contributions but no clear J:E interactions (F; 15 genes), and with strongly negative J:E interactions (G; 12 genes), respectively. Their heatmaps are shown. Rows in the heatmaps are scaled for better visualization using the most extreme value of each.

Since we observed concurrent spikes of JA and ET sector activity in our time course (S1 Fig), our dataset is a natural place to test this set of candidate genes. Of the 37 candidate ERF-branch genes whose transcriptional response was stronger with a combined exogenous JA and ethephon treatment than with only exogenous JA treatment, 30 genes showed a significant transcript response change in our dataset. Nearly all of these 30 genes showed an unexpected lack of strongly positive J:E interactions (Fig 10A). Although positive JA sector contributions at 2 hpt may be the most consistent pattern across the 30 genes, overall no dominating signaling allocation pattern was observed. The canonical ERF-branch marker genes *PDF1*.*2a* and *PR3* were not included in this set as they did not have significant flg22-specific responses. (Fig 10B and 10C). Although *PR4* was included in the set of 30 genes, it also had a very limited flg22-specific response (Fig 10D). However, it is noteworthy that the basal transcript level of PDF1.2a was highly positively regulated by the J:E interaction (Fig 10B; *JEPS*, *JEpS*, *JEPs*, *JEps*, and *fls2* have clearly higher basal transcript levels than the others) while the transcript response was not. These results indicate that for the synergistic activation of the ERF branch by the JA and ET sectors, something else is also required, such as stronger activations of the sectors, longer durations of the sector activations, and/or some other signaling factor(s), which is missing in our experimental system.

As an alternative approach we selected flg22-inducible genes that show synergy between JA and ET (i.e., positive J:E interactions and non-negative JA and ET contributions) (Fig 10E). This 41-gene set was enriched with 9 genes with a GO biological process term of oxoacid metabolic process (7.2-fold enrichment, *P* = 6.8E-3). It will be interesting to investigate whether these genes with strong positive J:E interactions are regulated as ERF-branch response genes but more sensitively than the 30 putative ERF-branch response genes or whether they represent a different regulatory mechanism. The fact that none of the 41 genes had the canonical ERF binding motif, AGCCGCC, in either strand in the 1000-bp upstream region suggests a different regulatory mechanism. We also found 15 genes mainly regulated by the additive effects of the JA and ET single sectors (Fig 10F), which was enriched with 3 genes with a GO biological process term of aromatic amino acid family biosynthetic process (> 100-fold enrichment, *P* = 3.7E-3), and 12 genes with negative J:E interactions and non-negative JA and ET sector contributions (Fig 10G). These genes controlled by qualitatively different JA:ET interactions exemplify that interactions downstream of the JA-ET-PAD4-SA network are complex as well (Fig 7).

### The power and limitation of network reconstitution-based signaling allocation analysis and a perspective

Since the concept of network reconstitution, which interprets network behavior in a manner different from conventional genetics, is not commonly used, we discuss the power and limitations of the approach. In conventional genetics, the null mutant phenotype is compared to the wild-type phenotype, and the mutant phenotype is considered to be the opposite of the functional effect of the gene that is mutated. In network reconstitution, a highly impaired state of the network (ideally the completely impaired state) is considered the basal state, and the phenotype change that is caused by addition of a functional gene to the basal state is considered as the functional effect of the gene. These two approaches do not result in any different conclusions if the effects of multiple genes of interest are additive and no interactions among them are present. Genes that control different pathways in a signaling network with a tree-like structure without cross-talk are an example of this case. However, when interactions within the network are non-negligible, the conventional genetic approach fails to provide a simple mechanistic interpretation of the results because a single mutation knocks out not only the function of the gene itself but also the interactions with other genes in the network and because these multiple effects cannot be separated in a way that allows a mechanistic interpretation. On the other hand, the network reconstitution approach provides a simple mechanistic interpretation of both the gene effects and their interactions among the genes included in the analysis. Complex behavior of a signaling network stems from signal convergence (cross-talk, feedback, feedforward, etc.) [50]. The plant immune signaling network is particularly highly interconnected [51] and has complex network properties, such as resilience and tunability. This is the reason network reconstitution is very useful in mechanistic investigations of the plant immune signaling network. A comparison of the PAD4 sector response time-course data in S1 Fig, panel F and the corresponding signaling allocation results in Fig 5C is a good demonstration of the power of network reconstitution. They are just different representations of the same time-course data obtained from the comprehensively combinatorial genotypes. The actual data in S1 Fig, panel F is visually uninterpretable regarding how the signaling sectors control the PAD4 sector response. With the concept of network reconstitution, signaling allocation analysis allows mechanistic interpretations of the data by decomposing them into the contributions of the signaling sectors and their interactions (Fig 5C).

It should be emphasized that the network reconstitution approach was made practical by network reduction to a network of four signaling sectors. Simultaneous impairment of all four signaling sectors almost completely abolishes flg22-PTI, which justifies use of the *quad* mutant state as the basal state in network reconstitution. By reducing the network components to perturb to four, it was feasible to generate Arabidopsis genotypes for all 16 possible combinatorial perturbations of the network. The comprehensive combinatorial perturbations were the key to empower network reconstitution analysis. Until recently, preparing such combinatorial genotypes seemed impractical except in powerful model genetic organisms, such as Arabidopsis. With current advances in efficient genome engineering using CRISPR/Cas9 [52], however, a network reconstitution approach should now be feasible in other plant species and other organisms.

One limitation of network reconstitution is related to its fundamental assumption. For interpretations of network reconstitution results, the network structure is assumed not to be significantly changed by the signaling sector state except for the parts directly connected to or from the perturbed sectors. This assumption is by no means guaranteed in a given network. Previously, we showed that it is not necessary to assume structural differences in the network to explain different network responses to different MAMPs upon network reconstitution [18]. In the current study, we showed that a significant change in wiring from the receptor FLS2 to the rest of the network is unlikely under our experimental conditions. Thus, we so far have not detected a major violation of this assumption in the plant immune signaling network consisting of the four signaling sectors.

Other limitations are not due to the conceptual framework of network reconstitution but are related to our current form of its implementation, signaling allocation analysis. Signaling allocation is a mechanistic description of a network’s behavior, and, thus, it allows mechanistic interpretations, which presents advances in the study of network properties as discussed above. Yet, it is not a mechanistic model: a mechanistic model must be a quantitative function that specifies the output signal based only on multiple input signals and the current state at each signaling sector. One reason signaling allocation is still a long way from building a mechanistic model is that the states of the signaling sectors as inputs to other sectors or to downstream transcriptional regulation are binary, wild type or mutant. To enable mechanistic modeling, the quantitative function at each signaling sector, including how multiple inputs that may be associated with some delays quantitatively interact in controlling the sector activity, needs to be learned from data. Network reconstitution was an essential part of collecting powerful data from a highly resilient network. Still, the statistical power of our data generated only with a single concentration of flg22 was not sufficiently high and limited us to a binary-input-based signaling allocation analysis. In the future, data need to be collected with various network input patterns to different signaling sectors with respect to the intensity, combination, and timing of these inputs, in conjunction with network reconstitution, to support mechanistic modeling.

### Conclusions

By definition, the components of a complex network strongly interact. In this paper we reduced a large complex network down to a manageable size—four major signaling sectors—to comprehensively probe, via network reconstitution, the sector interactions that regulate the transcriptome changes induced by a potent MAMP, flg22. Dynamic profiling of the transcriptome and two major immune hormones, SA and JA, across our set of comprehensive perturbation genotypes allowed us to quantitatively describe complex mechanisms that drive the flg22 transcriptome response. Our analysis revealed that regulation of the vast majority of flg22-responsive and network-dependent genes rely on interactions among the JA, ET, PAD4, and SA signaling sectors. In many cases, such sector interactions provide a basis for network buffering, and in several cases the sector interactions driving transcript expression were unexpected based on prior knowledge. Our work shows that high-order perturbation at the scale of signaling sectors and dynamic transcriptional profiling together can help in deciphering the regulation of a complex network, the plant immune signaling network. Network reconstitution is a powerful approach to reveal mechanisms that underlie the function of a complex biological network, especially one whose function is highly resilient against perturbations.

## Materials and methods

(See S1 Text for detailed method descriptions.)

### Plant materials, growth conditions, leaf tissue treatment and collection

Combinatorial mutants [11] of the *Arabidopsis thaliana* mutants *dde2-2* [14], *ein2-1* [15], *pad4-1* [16], and *sid2-2* [17] and the *fls2* mutant (SAIL_691_C4) [53], all in the *Col-0* background, were grown under a 12 hr photoperiod in a controlled environment at 22°C and 75% relative humidity. 3 leaves (leaf stages 7–9) of a single plant of each genotype from 31–32 days-old plants were syringe-inoculated with a 1 μM solution of flg22, and harvested at the indicated time after treatment. Different time point samples were collected from different plants. 0 h samples were untouched. For each biological replicate, leaves of plants from four independent experiments (3 leaves per plant ∙ 4 plants = 12 leaves in total) were pooled. Three biological replicates, from 12 independent experiments (4 plants per replicate ∙ 3 replicates = 12), were profiled for each genotype:time combination, for a total of 17 genotypes∙7 time points ∙3 biological replicates = 357 tissue samples. (See S3 Text for detailed planting, treatment, and harvesting information.) Tissue was flash frozen using liquid N_2_, powderized, and freeze-dried. Each tissue sample was divided for hormone measurement and for RNA extraction for Tag-Seq library preparation. The RNA samples used in this study for two of the seven time points, 3 and 9 hours post flg22 treatment, were exactly the same as those used in [18].

### Hormone measurement

Hormone extraction and concentration measurements were performed with an ultra-performance liquid chromatography-tandem mass spectrometer (UPLC-MS/MS) (ACQITY UPLC System/Quattro Premier XE; Waters) with an ODS column (ACQUITY UPLC BEH C18, 1.7 μm, 2.1 × 100 mm; Waters) as described previously [21,22]. Samples were processed in two separate batches. The SA and JA level raw data at 2 of 7 time points, 3 and 9 hpt, were the exactly same data used in [18]. Except for JA, the mean and standard error values for each genotype:time combination were estimated by fitting a linear model to the log_2_-transformed measured values for each hormone or related compound: *log*_2_[*hormone*] ~ *genotype*: *time*. We noticed that the JA measurement had substantial technical error when the JA level was very low since the log_2_-transformed measured values from the *dde2*-containing genotypes, in which the actual JA level is 0 [54], ranged from 4.0 to 11.4 (mean 7.6, standard deviation 1.6). We modified the very low levels of JA level values to moderate this issue, which are referred to as “adjusted log_2_ JA” values. See S1 Text for the detail.

### Tag-Seq library preparation and measurement of gene transcript expression levels

Stranded mRNA Tag-Seq libraries were prepared as previously described [20]. Internal barcodes were used to multiplex 16 different samples into a single lane of an Illumina flowcell, and sequenced using Illumina’s Genome Analyzer IIx System. Only sequencing reads with exact barcode matches were used. Barcodes were trimmed, and reads were uploaded to Galaxy [55–57]. Sequences that were mainly homopolymer sequence were removed using the Artifacts Filter in the fastx toolkit that is built in to Galaxy [58]. Remaining reads were mapped to TAIR10 transcripts using Bowtie (version bowtie-0.12.8) [59]. Note that sequence reads from the Tag-Seq method are concentrated in the 3’- regions of the transcripts. Mapping files for reads mapped to the forward strand were downloaded from Galaxy. Reads mapping to only a single gene were selected, and reads mapping to any transcript of a gene were summed together into a single gene-based count value. 26275 genes had at least one read count of 1 or higher (Fig 1). For each library, the ratio of the 90^th^ percentile read count value to 300 counts was log_*e*_-transformed and used as the *offset* in the negative binomial generalized linear model (glm-nb) below for the purpose of between-libraries normalization.

We filtered the genes to remove those that mostly have low transcript levels. First, genes with 50 or more read count values of 0 among the 357 values of the gene were removed. Second, genes whose 15^th^ count value from the top was 25 or lower were removed. After the first and second filtering, 18750 genes remained (Fig 1). The read count values of 0 in the data were floored as glm-nb does not behave well with 0 read counts (see S1 Text for details). The processed data were used to fit the glm-nb, *read count* ~ *genotype*: *time* − 1 + *offset*, for each of 18750 genes. The mean and standard error values were estimated in the log_*e*_ scale for every genotype:time combination in glm-nb for each of 18750 genes. The estimated values in the log_*e*_ scale were converted to the conventional log_2_ scale. The mean estimates in log_2_ were referred to as the transcript level, unless stated otherwise.

### Gene selection

To find genes significantly regulated by the JA-ET-PAD4-SA network, *p*-values from the glm.nb were calculated for 90 difference-in-differences values per gene: (*e*_*g*,*t*_ − *e*_*g*,*t* = 0_) − (*e*_*q*,*t*_ − *e*_*q*,*t* = 0_) where *e* is transcript mean estimate, *g* is any non-quadruple combinatorial mutant (15 available), *t* is a time point after treatment (1, 2, 3, 5, 9 or 18 hpt) and *q* is the quadruple mutant genotype. These difference-in-differences values are called transcript response changes here. The *p*-values (15 comparisons ∙ 6 time points = 90 comparisons in total) from the 18750 genes were calculated using *z*-test with the means and standard errors for the *e* values involved in the comparisons. The *p*-values for the same comparisons with hormones were also calculated similarly, except that *t*-test (2-sided test) was used instead of *z*-test. All *p*-values were corrected together for multiple-hypothesis testing using Storey’s FDR [60,61]. *q*-values (corrected *p*-values) less than 0.05 and transcript response changes that are greater than 2-fold or less than 0.5-fold were called significant. Genes with at least one significant transcript response change were considered significantly regulated by the network. For the cases (Fig 2 columns a and b; Fig 4) where genes were selected by transcript responses relative to wild type, the wild type genotype was used as the baseline in the transcript response change calculations, instead of the quadruple mutant.

### Signaling allocation models

For genes with significant transcript response changes, signaling allocation models were fit individually to each gene and hormone. These models were fit to transcript read count values or hormone abundance relative to steady state, *e*_*g*,*t*_ − *e*_*g*,*t* = 0_, where *g* represents genotype, *t* time, and *e* transcript expression level (or, for hormones, abundance) (see S1 Text for details). The design matrix of the models was made so that the signaling allocation variables (Table 1 in Tsuda et al., 2009 [11]) are fit to the transcript/hormone response, *e*_*g*,*t*_ − *e*_*g*,*t* = 0_. Each starting model was regularized (that is, made sparse) using lasso [25,26]. The glmregNB function in the mpath package and the lars function in the lars package were used for the transcript read counts and hormone abundance, respectively. The lasso penalty parameter that minimized fit AICc [27,28] was selected. Then glm-nb for the transcripts and an ordinary linear model for the hormones were re-fit using the selected coefficient subset, to avoid the coefficient shrinkage inherent in lasso. For S3 Fig, the starting (unregularized) models and the models regularized by the lasso parameter selected based on BIC [27], instead of AICc, were used.

### Clustering

The heatmaps and associated dendrograms in all Figs were produced using cosine distance complete-linkage agglomerative hierarchical clustering, except for S2 Fig, in which the Pearson correlation with the reversed sign was used as the distance metric.

### Gene Ontology (GO) enrichment analysis

GO enrichment analysis was performed using The Gene Ontology Consortium’s web-based application [62,63]. GO Ontology database release 2015-08-06; PANTHER Overrepresentation Test release 20150430.

## List of the AGI codes for the Arabidopsis genes

The following is the AGI codes of the Arabidopsis genes that are specifically described by their common names in the main text: *DDE2* (AT5G42650), *EIN2* (AT5G03280), *PAD4* (AT3G52430), *SID2* (AT1G74710), *FLS2* (AT5G46330), *ARGOS* (AT3G59900), *PR1* (AT2G14610), *NPR1* (AT1G64280), *MKK2* (AT4G29810), *EDS1* (AT3G48080), *SARD1* (AT1G73805), *PDF1*.*2a* (AT5G44420), *PR3* (AT3G12500), *PR4* (AT3G04720), and *ORA59* (AT1G06160).

### Data submission to NCBI’s gene expression omnibus

Tag-Seq data and the derived read-counts-per-gene data have been submitted to NCBI’s Gene Expression Omnibus (GEO); GEO data series GSE78735.

## Figshare information

Some of supplementary information is available from Figshare with a title of “Arabidopsis transcriptome/hormone response to flg22” and the following description [64]:

The raw data used to generate the data in this set: (GEO GSE78735) Arabidopsis thaliana RNA-3seq data collected from 17 genotypes (JEPS, jEPS, JePS, JEpS, JEPs, jePS, jEpS, jEPs, JepS, JePs, JEps, jepS, jePs, jEps, Jeps, jeps, fls2) x 7 time points (0, 1, 2, 3, 5, 9, 18 hours after flg22 treatment) x 3 biological replicates.

Contents of this set:

All are tab-delimited text. The number of columns does not include the row name column (gene/hormone name).

1. The mean and standard error estimates for each combination of the genotype and the time (119 columns) for each of 18750 genes (rows).
  - Mean: “genotype.time.mean.estimates.txt”
  - Standard error: “genotype.time.std.err.txt”
2. The same as 1. but for 37 hormones (rows) and related compounds (hormones, hereafter). Note that for JA, all 8 j-containing genotypes (such as jEPS, jEps) were handled as a single genotype, so they have the same values for a single time point.
  - Mean: “hormone.genotype.time.mean.estimates.txt”
  - Standard error: “hormone.genotype.time.std.err.txt”
3. The transcript response change (difference in differences) for the comparisons of each of the 15 combinatorial genotypes (not including the jeps) vs. jeps at each of 6 time points (1, 2, 3, 5, 9, 18 hours; 15 * 6 = 90 columns), for 18750 genes (rows).
  - Mean: “did.vs.quad.txt”
  - Associated p-value: “did.pval.vs.quad.txt”
4. The transcript response change (difference in differences) for the comparisons of each of the 15 combinatorial genotypes (not including JEPS) vs. JEPS (WT) at each of 6 time points (90 columns), for 18750 genes (rows).
  - Mean: “did.vs.wt.txt”
  - Associated p-value: “did.pval.vs.wt.txt”
5. The transcript response change (difference in differences) for the comparisons between fls2 and JEPS (WT) at each of 6 time points (6 columns), for 18750 genes (rows).
  - Mean: “did.fls2.vs.wt.txt”
  - Associated p-value: “did.pval.fls2.vs.wt.txt”
6. The same as 3., but for 37 hormones
  - Mean: “hormone.did.vs.quad.txt”
  - Associated p-value: “hormone.did.pval.vs.quad.txt”
7. The same as 5., but for 37 hormones
  - Mean: “hormone.did.fls2.vs.wt.txt”
  - Associated p-value: “hormone.did.pval.fls2.vs.wt.txt”
8. The signaling allocation results (16 allocations x 6 time points = 96 columns) with the model selected based on AICc. (used in most Figs for signaling allocations, including Fig 2 heatmap) for 5235 genes plus two hormones (rows).
  - Mean: “allocation.AICc.model.mean.txt”
  - Associated p-value: “allocation.AICc.model.pval.txt”
9. The signaling allocation results (16 allocations x 6 time points = 96 columns) with the full (unregularized) model. (used in the Fig 3 and S3A Fig) for 5259 genes plus two hormones (rows).
  - Mean: “allocation.full.model.mean.txt”
  - Associated p-value: “allocation.full.model.pval.txt”
10. The signaling allocation results (16 allocations x 6 time points = 96 columns) with the model selected based on BIC. (used in S3B Fig) for 4954 genes plus 2 hormones (rows).
  - Mean: “allocation.BIC.model.mean.txt”
  - Associated p-value: “allocation.BIC.model.pval.txt”
11. “genes.for.allocation.figs.txt”: The gene sets used in the figures indicated below among the genes in 8. (5235 genes plus 2 hormones; rows). A value of 0 indicates not used in the figure. For clusters and randomly selected genes, a value of 1 indicates the membership. For heatmaps, non-zero values indicate the order of the genes from the top in the figure. The figures are indicated in the column (22 columns): 'Fig2.heatmap', 'Fig2.column.a', 'Fig2.column.b', 'Fig6A.heatmap', 'Fig6B.heatmap', 'Fig6C.heatmap', 'Fig8.heatmap', 'Fig8.clusterS1', 'Fig8.clusterS2', 'Fig8.clusterS3', 'Fig9A.heatmap', 'Fig9B.heatmap', 'Fig9C.heatmap', 'Fig9A.clusterE1', 'Fig9A.clusterE2', 'Fig9A.clusterE3', 'Fig10A.heatmap', 'Fig10E.heatmap', 'Fig10F.heatmap', 'Fig10G.heatmap', 'S4Fig.heatmap', 'S7Fig.ET.geneset'
12. “S2Fig.genes.txt”: The gene sets used in the S2 Fig (panels A and B; 2 columns) among the genes in 1. (18750 genes; rows). A value of 0 indicates not used in the figure. Non-zero values indicate the order of the genes from the top in the figure.
13. “cor.design.mat.txt”: The cosine correlation among the signaling allocation terms in linear model. The design matrix for only one time point was used.

## Supporting information

S1 Data Raw hormone data. They are in pmol/g dry weight. U.Q., under quantification limit.
(TXT)

S1 Text Supporting methods. (DOCX)

S2 Text Infiltration schedules. (ZIP)

S1 Fig The timecourses of the activity levels (A, C, E, and G) and responses (B, D, F, and H) of the JA (A and B), ET (C and D), PAD4 (E and F), and SA (G and H) signaling sectors in 17 genotypes. The lines for the genotypes are color coded for combinations of the active signaling sectors: JA (red), ET (yellow), PAD4 (green), and SA (blue), except for *JEPS* (wild type, black), *jeps* (quad, gray), and *fls2* (pink). See the legend on the right. The responses are calculated by subtracting the value for 0 hpt in each genotype for each sector. Some lines specifically discussed in the main text are labeled with the genotype names. Log_2_-transformed free JA and SA levels were used for the JA and SA sector activities, respectively, and log_2_-transformed transcript levels of the marker genes *ARGOS* and AT4G04500 were used for the ET and PAD4 sector activities, respectively.
(TIF)

S2 Fig The transcript levels of the flg22-resonsive, network-dependent (A, 5259 genes) or network-independent (B, 2659 genes) genes. The heatmaps after clustering based on the Pearson correlation coefficient are shown. The log_2_-transformed transcript level data from the genotypes *fls2*, *JEPS*, and *jeps* at all the time points were used. See Fig 1 for selection of the genes. For better visualization, the color intensity of each row is scaled for the maximum value.
(TIF)

S3 Fig Signaling allocations with different stringency of regularization. Heatmaps of the signaling allocations based on the models with no regularization (A) and with the regularization stringency selected by BIC (B) are shown. The orders of the genes in the rows of the heatmaps are the same as that in Fig 2 to facilitate comparisons among these two panels and the Fig 2 heatmap. Thin black lines in (B) show the genes in which no signaling allocations remained significant after stringent regularization. For better visualization, the color intensity of each row is scaled for the most extreme value of the row. “(C)-(E) None-zero signaling allocation values are compared pairwise among the unregularized, AICc-selected, and BIC-selected models by scatter plot with the density contour. The signaling allocation values for each gene/hormone were scaled so that the highest absolute value of the wild-type fitted transcript responses across the time points becomes 1. The correlations observed in the plots show conservation of the values across different stringency levels of regularization.
(TIF)

S4 Fig Genes selected for the SA single sector dominance are likely regularization artifacts. Heatmaps of the signaling allocations based on the AICc-selected regularization (A) and no regularization (B) for seven genes that passed the filter for the SA single sector dominance are shown. Since there is no evidently consistent pattern between (A) and (B), it is likely that the allocation results in (A) are artifacts of regularization. For better visualization, the color intensity of each row is scaled for the most extreme value of the row.
(TIF)

S5 Fig Signaling allocation for alternative ET and PAD4 sector activity markers, AT3G16530 (A) and AT4G21840 (B). Heatmaps for the signaling allocations are shown. The allocation patterns are similar to those of the ET and PAD4 sector activity markers of our selection, *ARGOS* and AT4G04500, respectively (Fig 5B and 5C). The allocations were scaled for visualization using the most extreme value in each panel.
(TIF)

S6 Fig *FLS2* transcript levels in the *ein2*-containing genotypes. *FLS2* transcript levels in the *ein2*-containing genotypes are increased to nearly wild type levels within 1 hour after flg22 treatment. (A and B) Transcript level time courses of AT5G46330 (*FLS2*) for 17 genotypes are shown. The lines for the genotypes are color coded for combinations of the active signaling sectors: JA (red), ET (yellow), PAD4 (green), and SA (blue), except for *JEPS* (wild type, black), *jeps* (quad, gray), and *fls2* (pink). See the legend on the right. (B) A zoom-in of (A) on early time points. Note that lines for all *EIN2*-containing genotypes (lines containing yellow) are higher than the lines for all *ein2*-containing genotypes at 0 hpt but there is almost no difference between them at 1 hpt.
(TIF)

S7 Fig Transcript response timecourse comparisons of *ein2*-sensitive genes between wild type (*JEPS*) and *ein2* (*JePS*). (A) The timecourses of 100 genes randomly selected from 1270 genes whose transcript responses were significantly changed in *JePS* compared to *JEPS*. Each trace represents one of the 100 genes. The timecourse is color coded as shown. (B-E) Artificially generated examples of expected patterns in the timecourse comparison plot (A). The left panels show example transcript response timecourses in each of the genotypes *JEPS* (black) and *JePS* (red-green-blue). The right panels show what the timecourses in the left panel looks like in the timecourse comparison plot. If transcript response is delayed in *JePS* due to low FLS2 level at early time points, counter-clock-wise trace patterns (yellow to green to sky blue) are seen in the timecourse comparison plot as seen in (B-E, right panels). However, such trend is not evident in (A), indicating delayed transcript responses are not a major trend among these genes.
(TIF)

S8 Fig An early ethylene spike is evident with other ET marker genes. Mean transcript levels of common ET marker genes, across all genotypes and time points profiled. (A) *ARL* (AT2G44080) and (B) *EBF2* (AT5G25350). The lines for the genotypes are color coded for combinations of the active signaling sectors: JA (red), ET (yellow), PAD4 (green), and SA (blue), except for *JEPS* (wild type, black), *jeps* (quad, gray), and *fls2* (pink). See the legend on the right. Data were collected at 0, 1, 2, 3, 5, 9, and 18 hours after flg22 treatment.
(TIF)
