# Supplementary material for: The highly buffered Arabidopsis immune signaling network conceals the functions of its components
Source: PLoS Genet. 2017 May 4;13(5):e1006639. doi: 10.1371/journal.pgen.1006639 (PMC5417422; doi:10.1371/journal.pgen.1006639)
Supplement: S1 Text — (DOCX) [file pgen.1006639.s002.docx]

# Plant leaf tissue treatment and collection

To handle the large number of samples required, plants harvested at all needed time points could not be infiltrated all at the same time. Thus inoculation was staggered so that different timepoints were inoculated at different times. The infiltration times were arranged so that the collection times were, as much as was experimentally feasible, approximately the same time of day. Specifically, leaves for the 1, 2, 3, and 5 hr time points were all harvested one right after another within about 2 hours. The 0, 9 and 18 hr samples were harvested at other times of day. See S3 Text for detailed infiltration schedules for all experiments.

# Hormone measurement

## Modification of JA data

The log_2_-transformed measured values from the *dde2*-containing genotypes, in which the actual JA level is 0, ranged from 4.0 to 11.4 (mean 7.6, standard deviation 1.6). The log_2_-transformed measured values from the *DDE2*-containing genotypes ranged from 4.8 to 15.9. When the values from the *DDE2*-containing genotypes were fit to the above linear model, the estimated standard deviation of measurements for a single genotype:time combination was 0.86 in the log_2_ scale, indicating that the measured values for the JA level of 0 was abnormally over-dispersed. To avoid overestimation of the significance, we applied the following data modification for the very low JA level values (including 0). First, we designated the measured values “below detection” when log_2_(measured JA value) < 10.5. Second, the “below detection” values were substituted with log_2_-transformed values randomly sampled from *N*(8.8, 0.86^2^), in which the above estimated data standard deviation for the *DDE2*-containing genotypes was used. Third, the first and second steps were repeated 10 times to generate 10 datasets with randomly generated values for the very low JA level. Fourth, the linear model, ${log}_{2}\left[ JA \right] \sim genotype:time-1$, with the genotype factor in which all the *dde2*-containing genotypes combined to a single genotype, was fit to each of 10 generated datasets, and the mean and standard error estimates for each genotype:time combination were averaged across the 10 models. The obtained averaged mean and standard errors were used for “adjusted log_2_ JA” values.

**Fitting linear model to the data for hormones and related compounds, except for JA**

Seven among 43 hormones and related compounds (simply referred to as “hormones” hereafter; JA is not included) had 49 or more genotype:time combinations that had values under quantification (‘U.Q.’) for all three replicates and were removed. In the data for the remaining 36 hormones, ‘U.Q.’ values were converted to NA values, so that these values were ignored in the following linear model fitting. The linear model, ${log}_{2}\left[ hormone \right] \sim genotype:time-1$, was fit to the data for each of 36 remaining hormones, using the lm function in R. The obtained mean and standard error estimates were used for hormone selection below.

# Tag-Seq library preparation and measurement of gene transcript expression levels

Main text methods describe how raw gene transcript counts were tallied from sequencing data. See the NCBI Gene Expression Omnibus (GEO) Data Series GSE78735 for the sequencing data and sample metadata used in data processing. Tallied counts per gene in each sample are also available from this data series.

## Fitting the glm-nb to the Tag-Seq data

26,275 TAIR10 genes had at least one count in at least 1 library. Only these genes were used in further analyses. We used the R functions of glm.nb {MASS} and glmregNB {mpath} for the purpose of glm-nb fitting and the lasso regularization of glm-nb, respectively. For each library, the count value at the 90^th^ percentile *v_90_* was extracted. The log*_e_*-transformed ratio of *v_90_* from each library to 300 counts (log*_e_*(*v_90_*/300)) was used as the offset in the glm.nb and glmregNB functions, for the purpose of between-libraries normalization. The quartile values of log*_e_*(*v_90_*/300) across the libraries were:

0% 25% 50% 75% 100%

-2.407946 -1.529395 -1.261131 -1.069053 0.000000

We filtered the genes to remove those that mostly have very low transcript levels as described in the main text to obtain 18750 genes. Since the glm-nb does not behave well with 0 read count values, the data for 18750 genes were floored as follows. For the purpose of estimating the mean values for each genotype:time combination, when all count values from three replicates for the combination were 0, one of the three values which was from the library with the highest *v_90_* among the three libraries was changed to 1 (modified data 1). The reason the library with the highest *v_90_* was selected is that the value change of 0 to 1 has the least impact among the three replicate libraries. Since this data modification was to raise the lower boundary of the dynamic range, comparisons of these mean estimates to any higher mean estimates were more conservative than the true comparisons. While the mean estimates behave reasonably with at least one non-zero read count in each of the genotype:time combinations, the associated standard error was vastly overestimated if there was any 0 read count among the replicates of a genotype:time combination, which resulted in a large loss of power. To avoid this unreasonable loss of power, for the purpose of the standard error calculation, we used modified data, in which all 0 read count values were changed to 1 (modified data 2). Only the standard errors that are associated with 0 read counts in the modified data 1 were replaced with the standard error calculated with modified data 2.

The reason glm-nb was used is because the distribution of read count values for each genotype:time combination for each gene is approximated by random sampling from Poisson distributions whose mean values are sampled from a log-normal distribution since the expression levels of most genes are known to assume log-normal distributions (1). The negative binomial distribution can be defined as a Poisson-Gamma mixture, i.e., a distribution made by sampling from Poisson distributions whose mean values are sampled from a Gamma distribution. A log-normal distribution can be well approximated by a Gamma distribution, and thus the distribution of read count values can be well approximated by a negative binomial distribution. The mean and standard error values were estimated in the log*_e_* scale by the glm.nb function for every genotype:time combination for each of 18750 genes. The estimated values in the log*_e_* scale were converted to the conventional log_2_ scale by dividing them by log*_e_*2.

# Gene and hormone selection

## Calculation of transcript/hormone response changes and the associated p-values

A transcript response change is a difference in differences; it calculates the transcript response in a network reconstitution genotype relative to the transcript response in the null network genotype, the quadruple mutant *dde2 ein2 pad4 sid2*:

$r=\left( e_{g,t\neq0}- e_{g, t=0} \right)-\left( e_{q,t\neq0}- e_{q, t=0} \right)$

Where $r$ is the transcript response change, $e$ the mean transcript level, $g$ any combinatorial genotype other than $q$, the quadruple mutant.

**C**

For each gene, 90 transcript response change values were estimated, for 15 genotypes and 6 responsive time points. The standard error for the transcript response change, $w$*,* was calculated as follows.

$$w=\sqrt{\left( {s_{g,t\neq0}}^{2}+{s_{g, t=0}}^{2}+{s_{q,t\neq0}}^{2}+{s_{q, t=0}}^{2} \right)}$$

Where $s$ is the standard error associated with each of the mean transcript levels used in the transcript response change calculation above, and $g$ is any combinatorial genotype other than $q$, the quadruple mutant.

The *z*-value, $r/w$, was used in the *z*-test to calculate the *p*-value (2-sided test). The *p*-values for the same comparisons with hormones were also calculated similarly, except that *t*-test (2-sided test) was used instead of *z*-test. The residual degree of freedom from the model for each hormone was used as the degree of freedom in the *t*-test.

When genes/hormones were selected by transcript/hormone responses in *fls2* relative to wild type, the indices for $e$ and $s$ in the above formula for $r$ and $w$ were $g=fls2$ and $q=wild type$. When genes were selected by transcript responses relative to wild type, the wild type genotype was used as the base line instead of *quad* ($q=wild type$; Fig 2, columns a and b and Fig 4)

## Estimating q-values for transcript response changes

To select the flg22-responsive and network-dependent genes/hormones, 90 *p*-values from the comparisons of combinatorial genotypes to *quad* and 6 *p*-values from the comparisons of *fls2* to wild type for each of 18750 genes and 36 hormones except JA, or 48 *p*-values from the comparisons of combinatorial genotypes to *quad* (they were fewer because all *dde2*-containing genotypes were combined to *quad*) and 6 *p*-values from the comparisons of *fls2* to wild type for JA, were multiple tests corrected together (total 1803285 p-values since 225 *p*-values from the hormones were missing due to U.Q. values) using Storey’s FDR (2) . The qvalue function in the R package ‘qvalue’ (3) was used to apply Storey’s FDR. In any case when FDR was used in this study, the *p*-value distribution before correction was visually inspected in a histogram to assure that the *p*-value distribution satisfies an assumption of a homogenous null distribution for FDR. The *p*-value corrected by Storey’s FDR is designated the *q*-value. *q*-values less than 0.05 for transcript/hormone response changes greater than 1 or less than -1 (in log_2_ scale) in the wild type and *fls2* comparisons were considered significant for the flg22-responsiveness (7918 genes and SA). *q*-values less than 0.05 for transcript/hormone response changes greater than 1 or less than -1 (in log_2_ scale) in the combinatorial genotypes and *quad* comparisons were considered significant for the network dependence (9185 genes, SA, and JA). The intersect of these two sets, 5257 genes and SA were flg22-responsive, network-dependent genes/hormone. JA passed the hormone response change criteria but did not pass the flg22-specificity criterion as the JA level also strongly responded in *fls2* (S1 Fig, panel B). Nonetheless, we included JA in the subsequent analysis because many JA-responsive genes showed flg22-specific responses (see below) and because it is a well characterized immune hormone (4).

For the analyses in Fig 2 columns a and b, and Fig 4, gene set selection was based on transcript response changes relative to wild type for all 18750 genes, instead of relative to *quad*. The *p*-values for all comparisons between wild type and other combinatorial genotypes were corrected together by Storey’s FDR and used for the gene selections in the figures. For Fig 2 columns a and b and Fig 4, we were interested in genes without significant transcript response changes relative to wild type. Thus gene sets were selected as the complements of gene sets for significant difference, the latter of which had *q* < 0.05 for at least one of the transcript response changes relative to wild type greater than 1 or less than -1 (in log_2_ scale).

# Signaling allocation models

## Fitting signaling allocation models for transcript response change

For each gene, the glm-nb with the design matrix **M**, an offset vector $\boldsymbol{u}$ for the between-libraries normalization (a vector of log*_e_*(*v_90_*/300), which is the same for all the genes) and another offset vector $\boldsymbol{e}_{\boldsymbol{0}}$ for the mean estimates of the transcript level at *t* = 0 for the genotype same as that of the corresponding observation of the gene was fit.

$\boldsymbol{e}\sim M\boldsymbol{+offset}\left( \boldsymbol{u+}\boldsymbol{e}_{\boldsymbol{0}} \right)$ Model 1

Where $\boldsymbol{e}$ is a vector of read count values of length 288 (16 network reconstitution genotypes across 6 time points after 0h, with 3 biological replicates for each genotype:time factor combination),

$\boldsymbol{e}=\left[ \begin{matrix} \boldsymbol{e'}_{replicate 1} \\ \boldsymbol{e'}_{replicate 2} \\ \boldsymbol{e'}_{replicate 3} \end{matrix} \right]$

${\boldsymbol{e}^{\boldsymbol{'}}}_{replicate}\boldsymbol{=}\left[ \begin{matrix} \begin{matrix} \boldsymbol{\eta}_{\boldsymbol{t=1}} \\ \boldsymbol{\eta}_{\boldsymbol{t=2}} \\ \boldsymbol{\eta}_{\boldsymbol{t=3}} \end{matrix} \\ \boldsymbol{\eta}_{\boldsymbol{t=5}} \\ \begin{matrix} \boldsymbol{\eta}_{\boldsymbol{t=9}} \\ \boldsymbol{\eta}_{\boldsymbol{t=18}} \end{matrix} \end{matrix} \right]$

$\boldsymbol{\eta}_{\boldsymbol{t}}\boldsymbol{=}\left[ \begin{matrix} \begin{matrix} \begin{matrix} \begin{matrix} \boldsymbol{JEPS}_{t} \\ \boldsymbol{jEPS}_{t} \\ \boldsymbol{JePS}_{t} \end{matrix} \\ \boldsymbol{JEpS}_{t} \\ \boldsymbol{JEPs}_{t} \end{matrix} \\ \boldsymbol{jePS}_{t} \\ \boldsymbol{jEpS}_{t} \end{matrix} \\ \begin{matrix} \boldsymbol{jEPs}_{t} \\ \boldsymbol{JepS}_{t} \\ \boldsymbol{JePs}_{t} \end{matrix} \\ \begin{matrix} \boldsymbol{JEps}_{t} \\ \boldsymbol{jepS}_{t} \\ \begin{matrix} \boldsymbol{jePs}_{t} \\ \boldsymbol{jEps}_{t} \\ \begin{matrix} \boldsymbol{Jeps}_{t} \\ \boldsymbol{jeps}_{t} \end{matrix} \end{matrix} \end{matrix} \end{matrix} \right]$

Where genotypes are indicated by the presence ($\boldsymbol{J, E, P, S}$) or absence ($\boldsymbol{jeps}$) of the four sectors (JA, ET, PAD4, SA).

$\boldsymbol{a}$ is the coefficient vector for the design matrix M (thus it is not seen in Model 1), which is the signaling allocation:

$\boldsymbol{a=}\left[ \begin{matrix} \begin{matrix} \boldsymbol{\alpha}_{\boldsymbol{t=1}} \\ \boldsymbol{\alpha}_{\boldsymbol{t=2}} \\ \boldsymbol{\alpha}_{\boldsymbol{t=3}} \end{matrix} \\ \boldsymbol{\alpha}_{\boldsymbol{t=5}} \\ \begin{matrix} \boldsymbol{\alpha}_{\boldsymbol{t=9}} \\ \boldsymbol{\alpha}_{\boldsymbol{t=18}} \end{matrix} \end{matrix} \right]$

$\boldsymbol{\alpha}_{\boldsymbol{t}}\boldsymbol{=}\left[ \begin{matrix} \begin{matrix} \begin{matrix} \begin{matrix} \boldsymbol{JA}_{t} \\ \boldsymbol{ET}_{t} \\ {\boldsymbol{PAD}\boldsymbol{4}}_{t} \end{matrix} \\ \boldsymbol{SA}_{t} \\ \boldsymbol{J:E}_{t} \end{matrix} \\ \boldsymbol{J:P}_{t} \\ \boldsymbol{J:S}_{t} \end{matrix} \\ \begin{matrix} \boldsymbol{E:P}_{t} \\ \boldsymbol{E:S}_{t} \\ \boldsymbol{P:S}_{t} \end{matrix} \\ \begin{matrix} \boldsymbol{J:E:P}_{t} \\ \boldsymbol{J:E:S}_{t} \\ \begin{matrix} \boldsymbol{J:P:S}_{t} \\ \boldsymbol{E:P:S}_{t} \\ \begin{matrix} \boldsymbol{J:E:P:S}_{t} \\ \boldsymbol{remainder}_{t} \end{matrix} \end{matrix} \end{matrix} \end{matrix} \right]$

$M$ in model 1 is a block matrix, composed of three $M^{'}$matrices, one for each biological replicate:

$M=\left[ \begin{matrix} M' \\ M' \\ M^{'} \end{matrix} \right]$

where $M^{'}$is the block matrix:

$M^{'}= \left[ \begin{matrix} \begin{matrix} A & 0 & 0 \\ 0 & A & 0 \\ 0 & 0 & A \end{matrix} & \begin{matrix} 0 & 0 & 0 \\ 0 & 0 & 0 \\ 0 & 0 & 0 \end{matrix} \\ \begin{matrix} 0 & 0 & 0 \\ 0 & 0 & 0 \\ 0 & 0 & 0 \end{matrix} & \begin{matrix} A & 0 & 0 \\ 0 & A & 0 \\ 0 & 0 & A \end{matrix} \end{matrix} \right]$

where 0 represents a block of zeros, and A the signaling allocation matrix defined in (5):

$$A=\left[ \begin{matrix} 1 & 1 & 1 & 1 & 1/6 & 1/6 & 1/6 & 1/6 & 1/6 & 1/6 & 1/4 & 1/4 & 1/4 & 1/4 & 1 & 1 \\ 0 & 1 & 1 & 1 & 0 & 0 & 0 & 1/3 & 1/3 & 1/3 & 0 & 0 & 0 & 1 & 0 & 1 \\ 1 & 0 & 1 & 1 & 0 & 1/3 & 1/3 & 0 & 0 & 1/3 & 0 & 0 & 1 & 0 & 0 & 1 \\ 1 & 1 & 0 & 1 & 1/3 & 0 & 1/3 & 0 & 1/3 & 0 & 0 & 1 & 0 & 0 & 0 & 1 \\ 1 & 1 & 1 & 0 & 1/3 & 1/3 & 0 & 1/3 & 0 & 0 & 1 & 0 & 0 & 0 & 0 & 1 \\ 0 & 0 & 1 & 1 & 0 & 0 & 0 & 0 & 0 & 1 & 0 & 0 & 0 & 0 & 0 & 1 \\ 0 & 1 & 0 & 1 & 0 & 0 & 0 & 0 & 1 & 0 & 0 & 0 & 0 & 0 & 0 & 1 \\ 0 & 1 & 1 & 0 & 0 & 0 & 0 & 1 & 0 & 0 & 0 & 0 & 0 & 0 & 0 & 1 \\ 1 & 0 & 0 & 1 & 0 & 0 & 1 & 0 & 0 & 0 & 0 & 0 & 0 & 0 & 0 & 1 \\ 1 & 0 & 1 & 0 & 0 & 1 & 0 & 0 & 0 & 0 & 0 & 0 & 0 & 0 & 0 & 1 \\ 1 & 1 & 0 & 0 & 1 & 0 & 0 & 0 & 0 & 0 & 0 & 0 & 0 & 0 & 0 & 1 \\ 0 & 0 & 0 & 1 & 0 & 0 & 0 & 0 & 0 & 0 & 0 & 0 & 0 & 0 & 0 & 1 \\ 0 & 0 & 1 & 0 & 0 & 0 & 0 & 0 & 0 & 0 & 0 & 0 & 0 & 0 & 0 & 1 \\ 0 & 1 & 0 & 0 & 0 & 0 & 0 & 0 & 0 & 0 & 0 & 0 & 0 & 0 & 0 & 1 \\ 1 & 0 & 0 & 0 & 0 & 0 & 0 & 0 & 0 & 0 & 0 & 0 & 0 & 0 & 0 & 1 \\ 0 & 0 & 0 & 0 & 0 & 0 & 0 & 0 & 0 & 0 & 0 & 0 & 0 & 0 & 0 & 1 \end{matrix} \right]$$

The resulting coefficients of Model 1 (i.e., $\boldsymbol{a}$) were used in S2 Fig, panel A (full model, unregularized). However, with only three times as many data points as coefficients fit, there were very few cases where any of these coefficients had significant p-values, even without correcting for multiple-hypothesis testing across all genes. Moreover, biologically, it is unlikely that all available signaling allocation contributions are used to regulate each gene. Therefore we sought sparse models that still have excellent predictive power.

Least-angle regression (lasso) regularization (6,7) provided a unique path for dropping model parameters. Lasso defined an order in which to drop parameters, which allowed us to avoid the combinatorial explosion of possible paths for randomly dropping parameters. Lasso achieves regularization by using the L^1^-norm times a shrinkage factor, λ to penalize ordinary least-squares regression. Introducing this penalty has the effect of driving less significant parameters to zero, while shrinking all coefficients.

Using the glmregNB function in the R package ‘mpath’, coefficients were extracted for each step along the lasso shrinkage path. An ordinary least-squares model was then refit using the glm.nb function for each step, containing only the parameters with non-zero coefficients at that step, and the AICc and BIC (8,9) of the refit sparse model were recorded. Then the regularized refit models corresponding to the minimum AICc were used unless stated otherwise. AICc is a form of Akaike’s Information Criteria (AIC) that is corrected for finite sample sizes; using AICc is a standard statistical approach for balancing model complexity with model fit. After our lasso- and AICc-based regression, the models for many genes contained coefficients with highly-significant *p*-values. For S3 Fig, panel B, the regularized refit models corresponding to the minimum BIC were used. BIC-selected regularization is in general more stringent than AICc-selected, and the numbers of non-zero parameters were lower.

# Selection of sector activity marker genes for the ET and PAD4 sectors

For selection of PAD4 sector activity markers, the following criteria were used (all transcript responses are in log_2_ scale): (1) for sufficiently precise measurements, the highest transcript response across the time points in wild type was higher than 2; (2) for the flg22-specificity, the difference in the transcript responses between wild type and *fls2* at the maximum time point in (1) was higher than 2; (3) for the *PAD4* specificity, the transcript response in each of the *pad4*-containing genotypes was less than half of the transcript response in the corresponding *PAD4*-containing genotypes; (4) for the unresponsiveness in all *pad4*-containing genotypes, the standard deviation of the transcript response across all *pad4*-containing genotypes and all time points are smaller than one fourth of the highest transcript response across all *PAD4*-containing genotypes and all time points. In this way, we identified AT4G04500 (our PAD4 sector activity marker) and AT4G21840 (S5 Fig, panel B). We visually inspected the transcript responses of these genes across the genotypes and the time points to make sure that the responses are consistently lower in the *pad4*-containing genotypes than the *PAD4*-containing genotypes.

Similarly, for selection of ET sector activity markers, the following criteria were used (all transcript responses are in log_2_ scale): (1) for sufficiently precise measurements, the highest transcript response across the time points in wild type was higher than 3; (2) for the flg22-specificity, the difference in the transcript responses between wild type and *fls2* at the maximum time point in (1) was higher than 1.5; (3) for the *PAD4* specificity, the transcript response in each of the *pad4*-containing genotypes was lower than two thirds of the transcript response in the corresponding *PAD4*-containing genotypes; (4) for the unresponsiveness in all *pad4*-containing genotypes, the standard deviations of the transcript responses across all *pad4*-containing genotypes and all time points are smaller than one fourth of the highest transcript response across all *PAD4*-containing genotypes and all time points. The threshold values of some of the criteria were changed from the PAD4 marker selection to obtain a reasonable number of candidates. Thirteen candidates were selected. We visually investigated the transcript responses of these genes and selected genes that have transcript responses consistently lower in the *pad4*-containing genotypes than the *PAD4*-containing genotypes across the time points. In this way, we selected AT3G59900 (*ARGOS*, our ET sector marker gene) and AT3G16530 (S5 Fig, panel A). The transcript levels of *ARL* (AT2G44080) and *EBF2* (AT5G25350), which are commonly used ET marker genes (10), were also used in S8 Fig to support the notion of early ET signaling after flg22 treatment.

**Selection of genes for figures.**

The JA single sector dominant genes in Fig 6A were selected for that the maximum absolute value of the JA single sector contributions across the time is at least 1.6-time greater than the maximum absolute value of all the other sector contributions and interactions across the time. The ET, PAD4, and SA single sector dominant genes in Fig 6B, Fig 6C, and S4 Fig, respectively, were selected similarly, but for each corresponding single sector contributions.

The apparent SA-responsive genes in Fig 8 were first selected for *q* < 0.05 and the absolute difference value in log_2_-scale > 1 (i.e., greater than 2-fold change) in all three criteria at at least one time point: (1) flg22-responsiveness in *JEPS* (WT), which was defined by differences at non-0 time points from 0 h in *JEPS*; (2) FLS2-dependence, which was defined by the difference between *fls2* and *JEPS* at each non-0 time point; (3) SA-dependence, which was defined by the difference between *JEPs* and *JEPS* at each non-0 time point. The *q*-value was calculated from the *p*-values for the three criteria combined. Additionally, the sign of the responsiveness, FLS2-dependence and SA-dependence were required to be the same (e.g., if it is flg22-inducible, the transcript levels were required to be lower in *fls2* and *JEPs* compared to *JEPS*). Among the selected, the genes that overlap with the genes in Fig 2 were shown in Fig 8. The apparent ET-, JA-, and PAD4-responsive genes in Figs 9A, 9B, and 9C were selected similarly, but for the corresponding single mutants, *JePS*, *jEPS*, and *JEpS*, respectively, instead of *JEPs*.

The genes with strong synergistic J:E interactions in Fig 10E were selected for: (1) the maximum absolute value of the J:E interactions across the time is greater than the maximum absolute value of the other sector contributions and interactions except for the JA and ET single sector contributions across the time; AND (2) at the time for the maximum absolute value of the J:E interactions, all of the J:E interactions and the JA and ET single sector contributions were non-negative. The genes with the JA and ET single sector additive contributions in Fig 10F were selected for: (1) at the time when the absolute value of the addition between the JA and ET single sector contributions was maximum, both of the JA and ET single sector contributions were positive; AND (2) both of the JA and ET single sector contributions at the same time were greater than the maximum absolute values of the other sector contributions and interactions across the time. The genes with strongly buffering J:E interactions in Fig 10G were selected for: (1) the maximum absolute value of the J:E interactions across the time is greater than the maximum absolute value of the other sector contributions and interactions except for the JA and ET single sector contributions across the time; AND (2) at the time for the maximum absolute value of the J:E interactions, the J:E interaction was negative, and the JA and ET single sector contributions were both positive.

# References

1. Bengtsson M, Ståhlberg A, Rorsman P, Kubista M. Gene expression profiling in single cells from the pancreatic islets of Langerhans reveals lognormal distribution of mRNA levels. Genome Res. 2005;15(10):1388–92.

2. Storey JD, Tibshirani R. Statistical significance for genomewide studies. Proc Natl Acad Sci U S A [Internet]. 2003 Aug;100(16):9440–5. Available from: http://www.pnas.org/content/100/16/9440.full

3. Storey JD. qvalue: Q-value estimation for false discovery rate control [Internet]. 2015. Available from: http://qvalue.princeton.edu/

4. Campos ML, Kang JH, Howe GA. Jasmonate-Triggered Plant Immunity. J Chem Ecol. 2014;40(7):657–75.

5. Tsuda K, Sato M, Stoddard T, Glazebrook J, Katagiri F. Network properties of robust immunity in plants. PLoS Genet. 2009 Dec;5(12).

6. Tibshirani R. Regression shrinkage and selection via the lasso: a retrospective. J R Stat Soc Ser B (Statistical Methodol [Internet]. 2011 Jun;73(3):273–82. Available from: http://doi.wiley.com/10.1111/j.1467-9868.2011.00771.x

7. Efron B, Hastie T, Johnstone I, Tibshirani R. Least angle regression. Ann Stat [Internet]. 2015 Dec;32(2):407–99. Available from: http://projecteuclid.org/euclid.aos/1083178935

8. Burnham KP. Multimodel Inference: Understanding AIC and BIC in Model Selection. Sociol Methods {&} Res [Internet]. 2004 Nov;33(2):261–304. Available from: http://smr.sagepub.com/content/33/2/261.short?rss=1%7B&%7Dssource=mfr

9. Hurvich CM, Tsai C-L. Regression and time series model selection in small samples. Biometrika [Internet]. 1989;76(2):297–307. Available from: http://www.scopus.com/inward/record.url?eid=2-s2.0-70349119250%7B&%7DpartnerID=tZOtx3y1

10. Guo H, Ecker JR. Plant Responses to Ethylene Gas Are Mediated by SCFEBF1/EBF2-Dependent Proteolysis of EIN3 Transcription Factor. Cell [Internet]. 2003 Dec;115(6):667–77. Available from: http://www.sciencedirect.com/science/article/pii/S0092867403009693
