# Supplementary figures and images for: The highly buffered Arabidopsis immune signaling network conceals the functions of its components
Source: PLoS Genet. 2017 May 4;13(5):e1006639. doi: 10.1371/journal.pgen.1006639 (PMC5417422; doi:10.1371/journal.pgen.1006639)

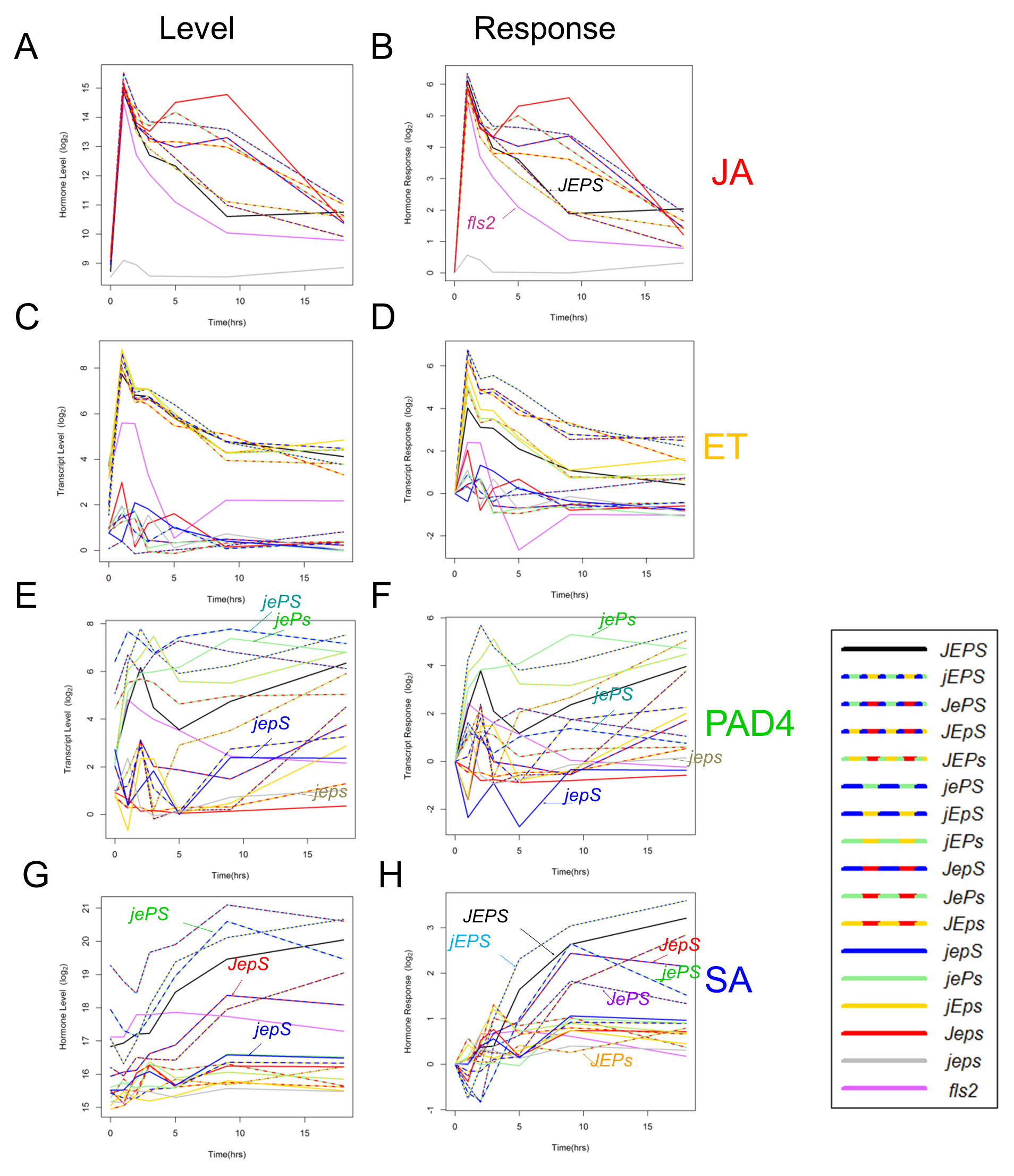

Supplement: S1 Fig — The lines for the genotypes are color coded for combinations of the active signaling sectors: JA (red), ET (yellow), PAD4 (green), and SA (blue), except for JEPS (wild type, black), jeps (quad, gray), and fls2 (pink). See the legend on the right. The responses are calculated by subtracting the value for 0 hpt in each genotype for each sector. Some lines specifically discussed in the main text are labeled with the genotype names. Log2-transformed free JA and SA levels were used for the JA and SA sector activities, respectively, and log2-transformed transcript levels of the marker genes ARGOS and AT4G04500 were used for the ET and PAD4 sector activities, respectively. (TIF) [file pgen.1006639.s004.tif]

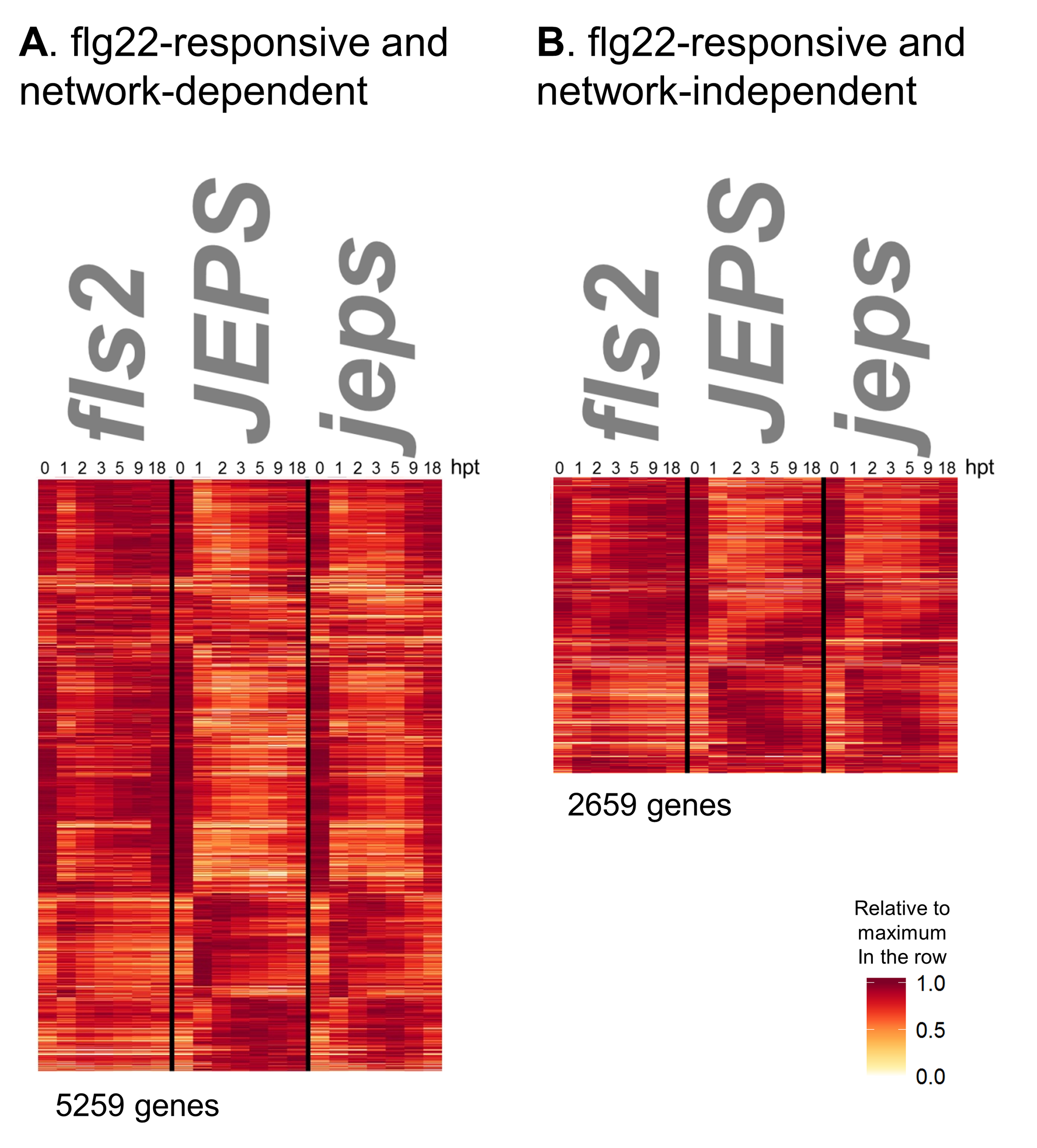

Supplement: S2 Fig — The heatmaps after clustering based on the Pearson correlation coefficient are shown. The log2-transformed transcript level data from the genotypes fls2, JEPS, and jeps at all the time points were used. See Fig 1 for selection of the genes. For better visualization, the color intensity of each row is scaled for the maximum value. (TIF) [file pgen.1006639.s005.tif]

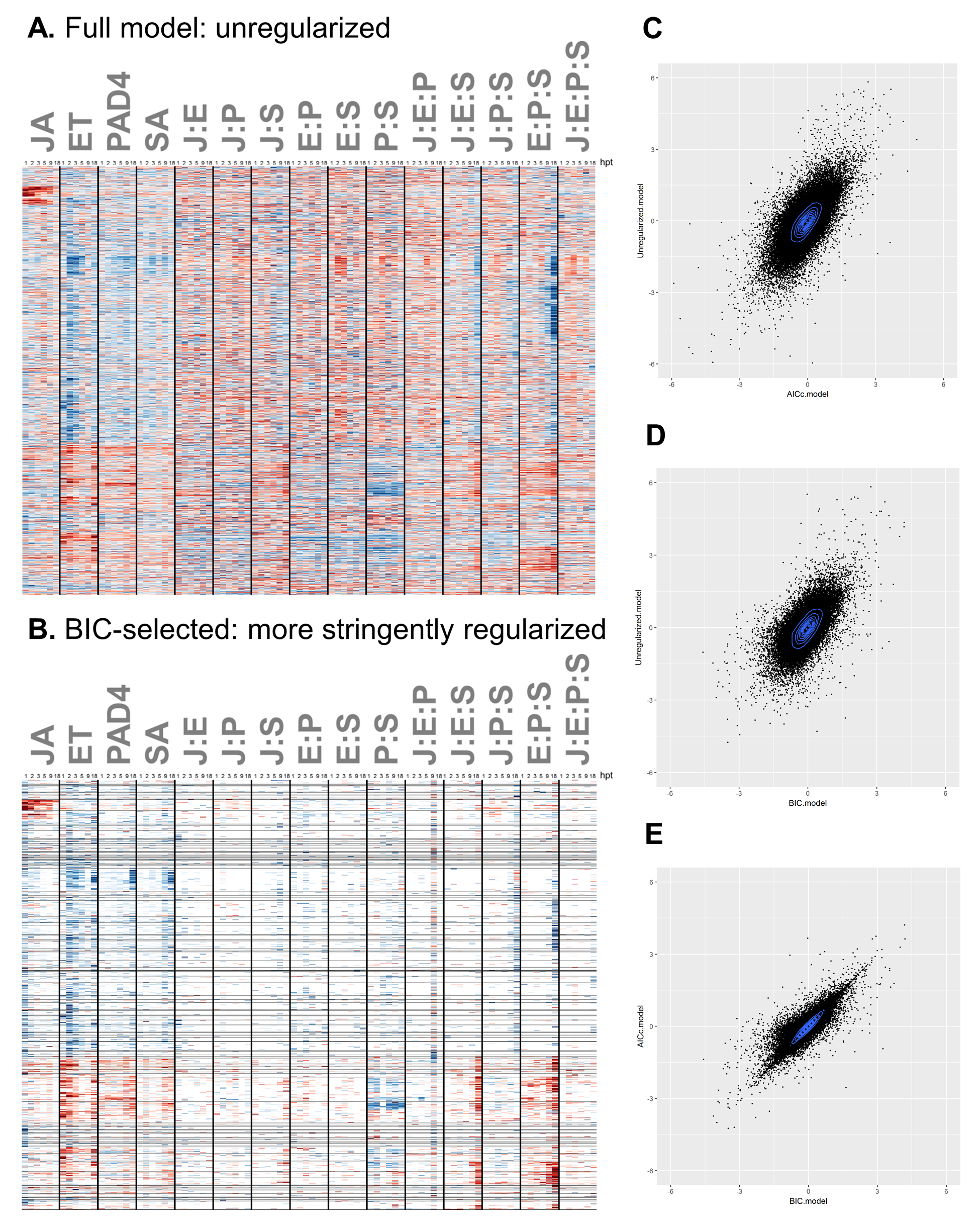

Supplement: S3 Fig — Heatmaps of the signaling allocations based on the models with no regularization (A) and with the regularization stringency selected by BIC (B) are shown. The orders of the genes in the rows of the heatmaps are the same as that in Fig 2 to facilitate comparisons among these two panels and the Fig 2 heatmap. Thin black lines in (B) show the genes in which no signaling allocations remained significant after stringent regularization. For better visualization, the color intensity of each row is scaled for the most extreme value of the row. “(C)-(E) None-zero signaling allocation values are compared pairwise among the unregularized, AICc-selected, and BIC-selected models by scatter plot with the density contour. The signaling allocation values for each gene/hormone were scaled so that the highest absolute value of the wild-type fitted transcript responses across the time points becomes 1. The correlations observed in the plots show conservation of the values across different stringency levels of regularization. (TIF) [file pgen.1006639.s006.tif]

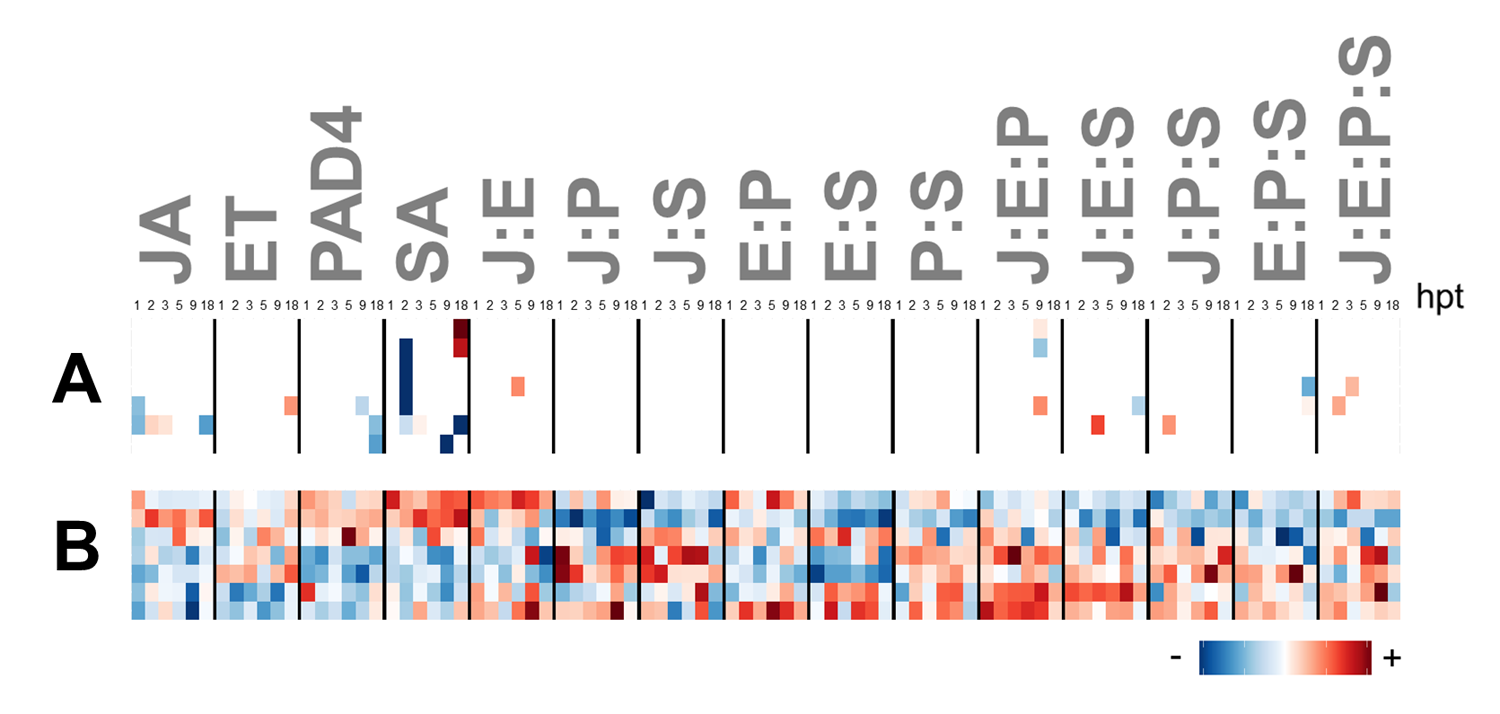

Supplement: S4 Fig — Heatmaps of the signaling allocations based on the AICc-selected regularization (A) and no regularization (B) for seven genes that passed the filter for the SA single sector dominance are shown. Since there is no evidently consistent pattern between (A) and (B), it is likely that the allocation results in (A) are artifacts of regularization. For better visualization, the color intensity of each row is scaled for the most extreme value of the row. (TIF) [file pgen.1006639.s007.tif]

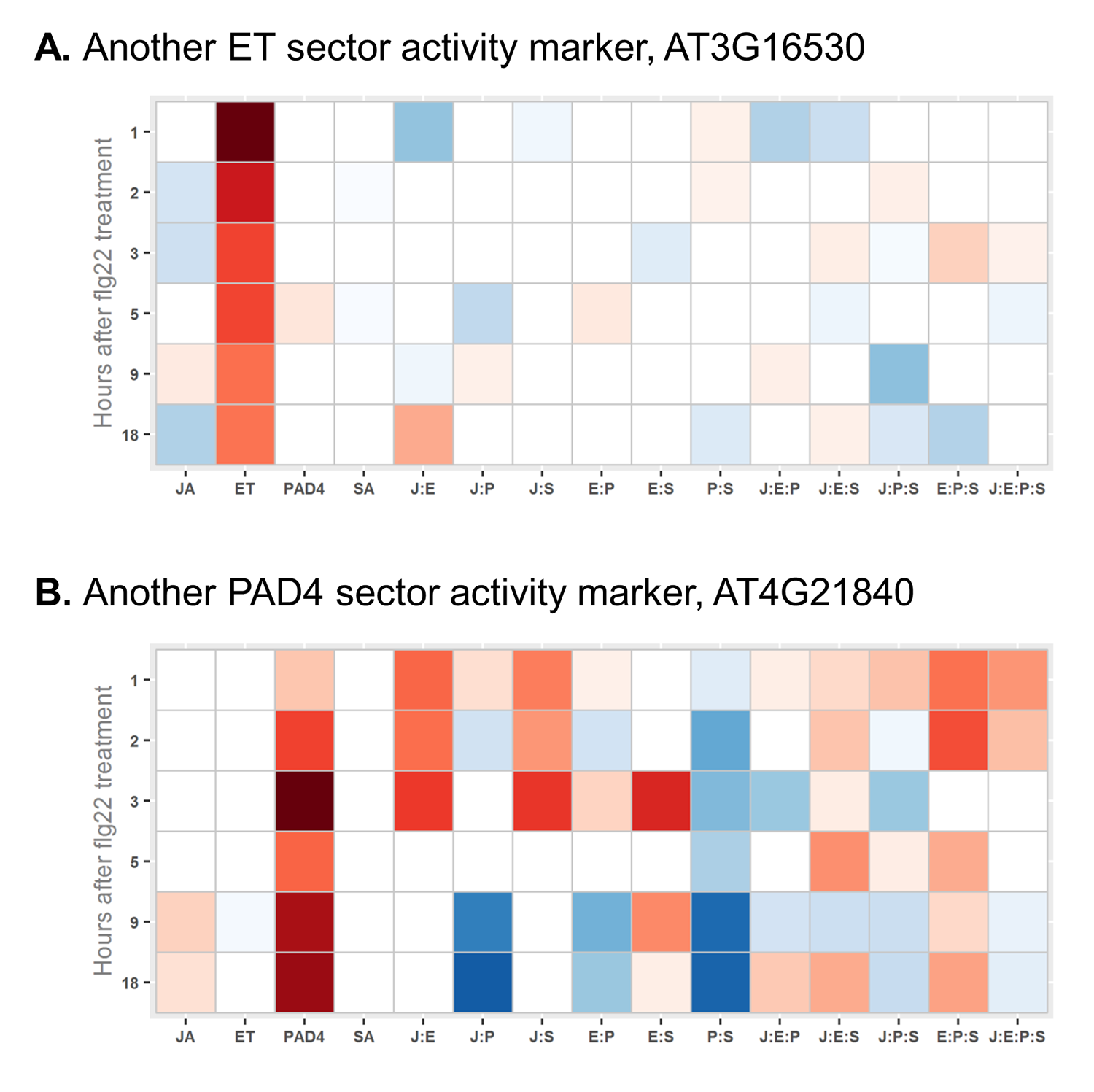

Supplement: S5 Fig — Heatmaps for the signaling allocations are shown. The allocation patterns are similar to those of the ET and PAD4 sector activity markers of our selection, ARGOS and AT4G04500, respectively (Fig 5B and 5C). The allocations were scaled for visualization using the most extreme value in each panel. (TIF) [file pgen.1006639.s008.tif]

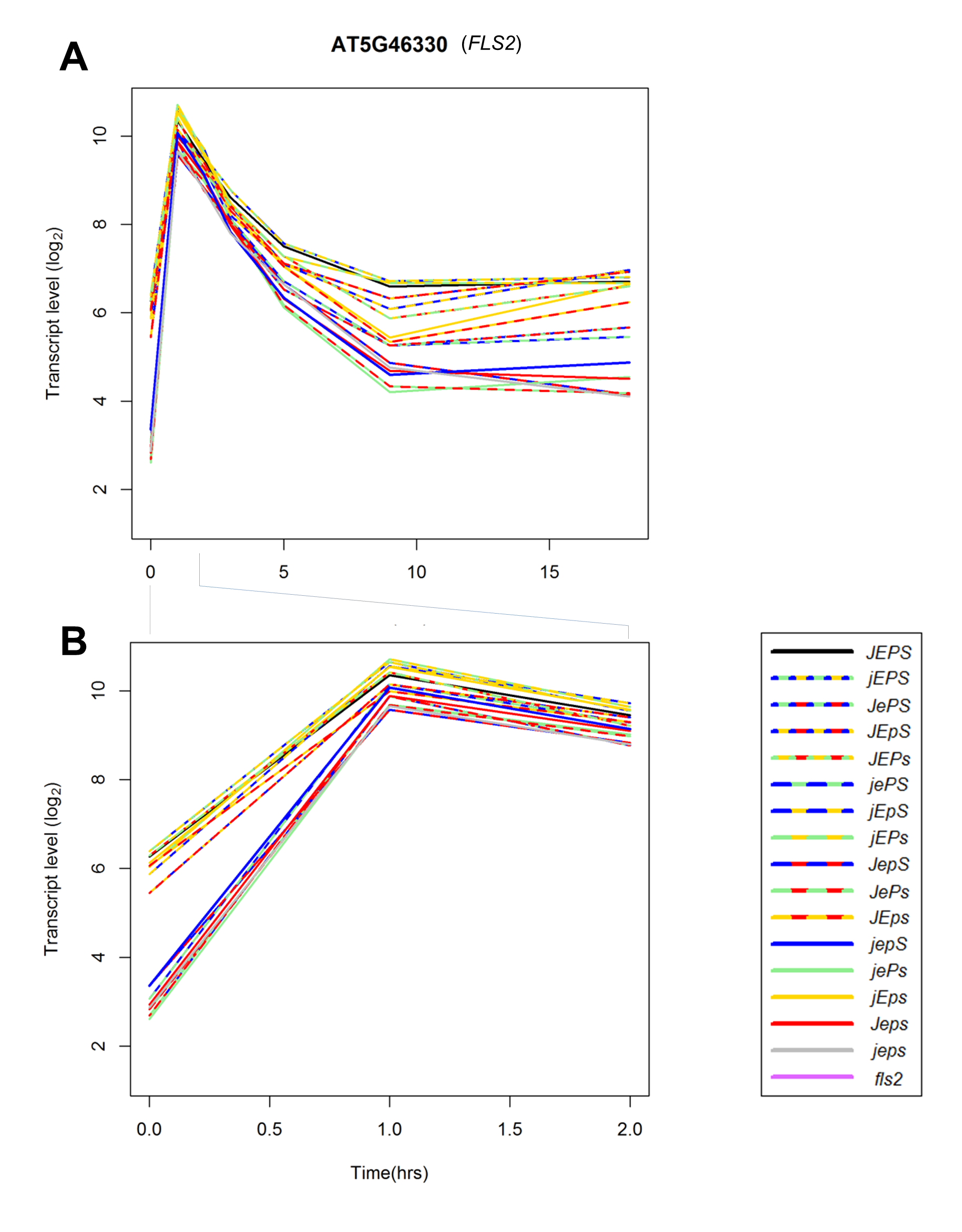

Supplement: S6 Fig — FLS2 transcript levels in the ein2-containing genotypes are increased to nearly wild type levels within 1 hour after flg22 treatment. (A and B) Transcript level time courses of AT5G46330 (FLS2) for 17 genotypes are shown. The lines for the genotypes are color coded for combinations of the active signaling sectors: JA (red), ET (yellow), PAD4 (green), and SA (blue), except for JEPS (wild type, black), jeps (quad, gray), and fls2 (pink). See the legend on the right. (B) A zoom-in of (A) on early time points. Note that lines for all EIN2-containing genotypes (lines containing yellow) are higher than the lines for all ein2-containing genotypes at 0 hpt but there is almost no difference between them at 1 hpt. (TIF) [file pgen.1006639.s009.tif]

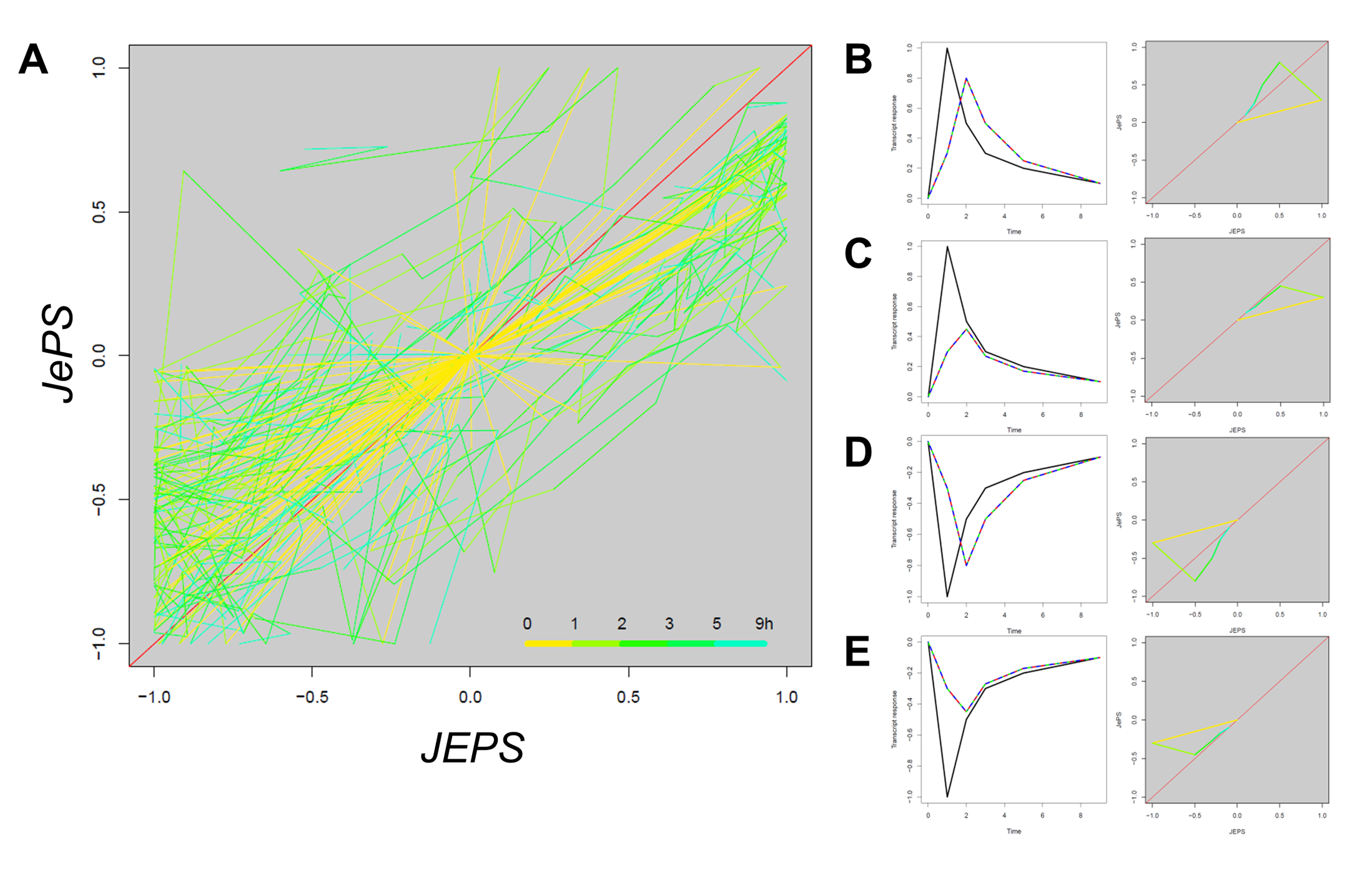

Supplement: S7 Fig — (A) The timecourses of 100 genes randomly selected from 1270 genes whose transcript responses were significantly changed in JePS compared to JEPS. Each trace represents one of the 100 genes. The timecourse is color coded as shown. (B-E) Artificially generated examples of expected patterns in the timecourse comparison plot (A). The left panels show example transcript response timecourses in each of the genotypes JEPS (black) and JePS (red-green-blue). The right panels show what the timecourses in the left panel looks like in the timecourse comparison plot. If transcript response is delayed in JePS due to low FLS2 level at early time points, counter-clock-wise trace patterns (yellow to green to sky blue) are seen in the timecourse comparison plot as seen in (B-E, right panels). However, such trend is not evident in (A), indicating delayed transcript responses are not a major trend among these genes. (TIF) [file pgen.1006639.s010.tif]

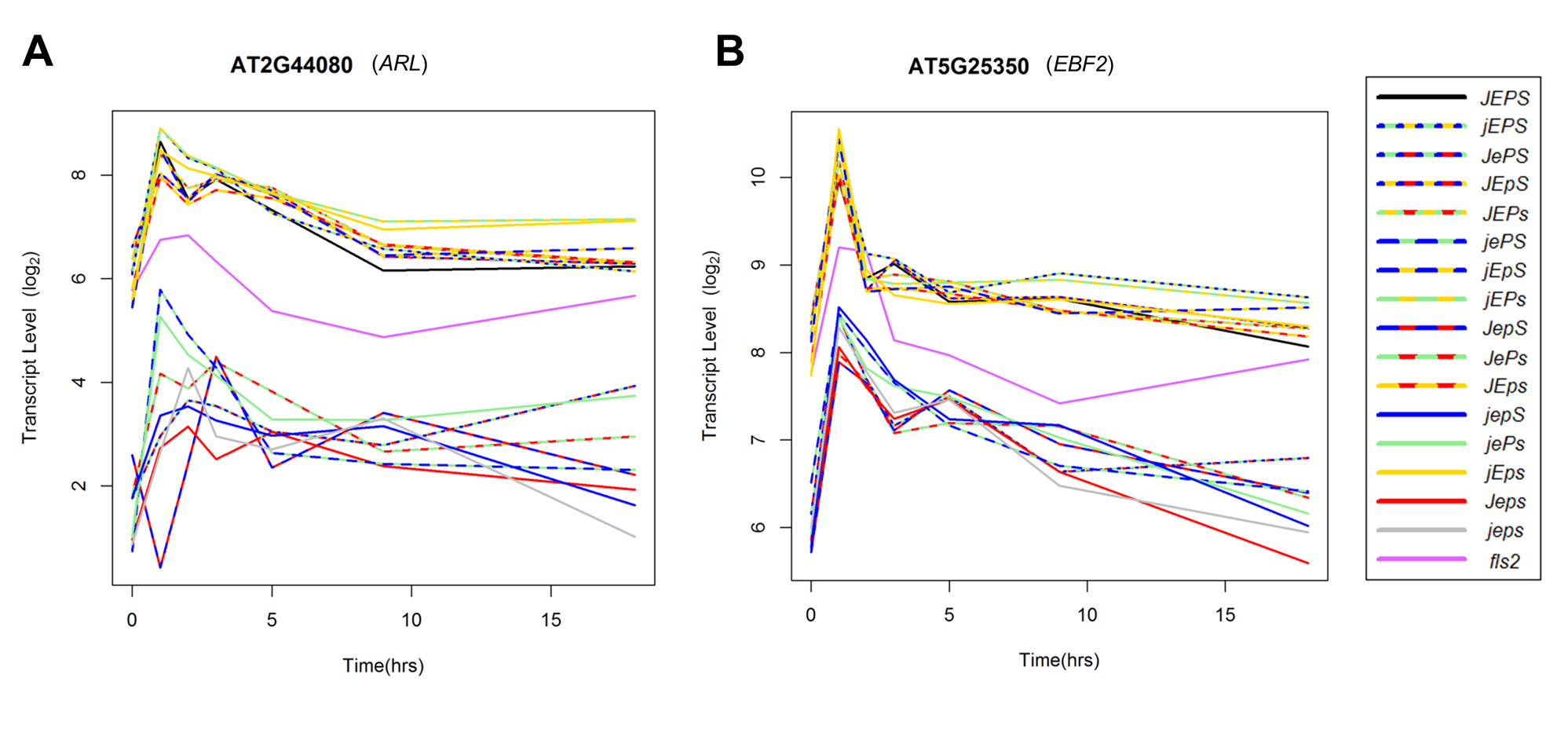

Supplement: S8 Fig — Mean transcript levels of common ET marker genes, across all genotypes and time points profiled. (A) ARL (AT2G44080) and (B) EBF2 (AT5G25350). The lines for the genotypes are color coded for combinations of the active signaling sectors: JA (red), ET (yellow), PAD4 (green), and SA (blue), except for JEPS (wild type, black), jeps (quad, gray), and fls2 (pink). See the legend on the right. Data were collected at 0, 1, 2, 3, 5, 9, and 18 hours after flg22 treatment. (TIF) [file pgen.1006639.s011.tif]
